# FISHing for ciliates: Catalyzed reporter deposition fluorescence *in situ* hybridization for the detection of planktonic freshwater ciliates

**DOI:** 10.3389/fmicb.2022.1070232

**Published:** 2022-12-12

**Authors:** Gianna Dirren-Pitsch, Dominique Bühler, Michaela M. Salcher, Barbara Bassin, Alizée Le Moigne, Martina Schuler, Jakob Pernthaler, Thomas Posch

**Affiliations:** ^1^Limnological Station, Department of Plant and Microbial Biology, University of Zurich, Kilchberg, Switzerland; ^2^Department of Aquatic Microbial Ecology, Institute of Hydrobiology, Biology Centre of the Czech Academy of Sciences, České Budĕjovice, Czechia

**Keywords:** planktonic ciliates, CARD-FISH, fluorescence *in situ* hybridization, lake water samples, quantitative protargol staining, ciliate quantification

## Abstract

Planktonic ciliate species form multiple trophic guilds and are central components of freshwater food webs. Progress in molecular analytical tools has opened new insight into ciliate assemblages. However, high and variable 18S rDNA copy numbers, typical for ciliates, make reliable quantification by amplicon sequencing extremely difficult. For an exact determination of abundances, the classical morphology-based quantitative protargol staining is still the method of choice. Morphotype analyses, however, are time consuming and need specific taxonomic expertise. Catalyzed reporter deposition fluorescence *in situ* hybridization (CARD-FISH) may represent a promising tool for the analysis of planktonic ciliates by combining molecular identification with microscopic quantification. We tested the applicability of CARD-FISH using nine cultured ciliate species. Eight species- and three genus-specific oligonucleotide probes were designed based on their 18S rRNA genes. The CARD-FISH protocol was adapted and the specificity of probes was established. We subsequently examined the precision of quantitation by CARD-FISH on single cultures and mock assemblages. Successful tests on lake water samples proved that planktonic ciliates could be identified and quantified in field samples by CARD-FISH. Double hybridizations allowed studying interspecific predator prey interactions between two ciliate species. In summary, we demonstrate that CARD-FISH with species-specific probes can facilitate studies on the population dynamics of closely related, small sized or cryptic species at high sampling frequencies.

## Introduction

Planktonic freshwater ciliates (Ciliophora) play a pivotal role in food webs of any standing water by interacting with multiple organismic entities in the plankton (Müller, 1989; Bandelt et al., 1999; Posch et al., 2015; Qu et al., 2021). Ciliate assemblages in freshwaters impact several trophic levels by being composed of particularly omnivorous but also bacterivorous, algivorous, and even predatory species (Weisse, 2017). Ciliates are, at least seasonally, a rich food source for rotifers, cladocerans, and copepods (Wickham, 1998; Agasild et al., 2012). The functional diversity is associated with a high taxonomic diversity found even in a single pond or lake. According to a compilation on planktonic ciliates (Foissner et al., 1999), there are at least 200 euplanktonic species temporarily colonizing the pelagial, reaching abundances between less than 1 and up to 1,000 individuals per milliliter (Müller et al., 1991a; Šimek et al., 2019; Posch et al., 2021).

It is a methodological challenge to analyze planktonic ciliate assemblages, i.e., to reach a high taxonomic resolution and to simultaneously get precise quantification of abundant as well as rare and ephemeral species using a single technique. For decades, microscopy of silver impregnated preparations represented a good compromise to fulfill both requirements. The quantitative protargol staining (QPS) has developed into a gold standard for the quantitative analysis of planktonic species (Montagnes and Lynn, 1987; Skibbe, 1994; Pfister et al., 1999). Main drawbacks of this approach are the required taxonomic expertise, the time-consuming assessment of the preparations, and the limited resolution of morphological details. Particularly the quantification and taxonomic distinction of tiny (<15 μm) and similar looking species is difficult. Ribosomal RNA-targeting molecular techniques have revolutionized the analysis of ciliate assemblages by their potential to detect rare, cryptic, and ephemeral species (Abraham et al., 2019). However, current techniques for next generation sequencing (NGS) of planktonic ciliates are not quantitative and cause artifacts with respect to *in situ* ciliate abundances (Pitsch et al., 2019; Santoferrara, 2019), e.g., due to multiple and taxonomically unevenly distributed copies of rRNA coding genes [up to thousands per individual ciliate (Gong et al., 2013)].

These shortcomings of NGS analysis can be overcome by the complementary use of microscopy-based molecular methods such as fluorescence *in situ* hybridization (FISH). It is based on the hybridization of fluorochrome-labeled oligonucleotide probes with a target rRNA sequence in an intact cell, resulting in signals which can be detected by fluorescence microscopy (Levsky and Singer, 2003). FISH thus combines the precision needed for determining species with the quantitative accuracy of microscopic counts of cells. Catalyzed reporter deposition FISH (CARD-FISH, Sekar et al., 2003), an improved version of FISH with enzymatic signal amplification, has become a widely recognized quantitative method for studying prokaryotic communities (Amann and Fuchs, 2008). Recently, Piwosz et al. (2021) have proposed CARD-FISH as a promising tool for the quantification of planktonic protists, especially of nanoplanktonic flagellates. The method allows to study protists by revealing their identity and feeding strategy, thus providing novel insights into their ecology (Piwosz and Pernthaler, 2010; Ballen-Segura et al., 2017; Grujcic et al., 2018; Šimek et al., 2021). So far, FISH has rarely been applied for the *in situ* quantification (Lim et al., 1996) or identification of ciliates (Fried et al., 2002) and most recent studies have focused on few economically important species in aquacultures (Zhan et al., 2014, 2018; Zhan and Xu, 2020). In a preliminary study, we observed that FISH with fluorescently monolabeled probes worked well in ciliate cultures. However, the high background of non-specific fluorescent signals from debris particles and other microorganisms prohibited a reliable detection and quantification of the hybridized cells in lake water samples by this approach (Bühler, 2020).

The aim of our study was thus to develop the first CARD-FISH protocol for the analysis of free-living planktonic ciliates and to test in detail its quantitative nature. Our methodological workflow included the sequencing of the 18S rDNA of ciliate species typically found in freshwater lakes and the design of corresponding oligonucleotide probes, followed by tests with cultured species and mock assemblages, and finally by the analysis of field samples. We emphasize the potential of this approach for future studies on planktonic freshwater ciliates, but also give caveats about possible artifacts and limitations when ‘FISH-ing for ciliates’.

## Materials and methods

### Origin and cultivation of studied ciliates

**Figure 1:**
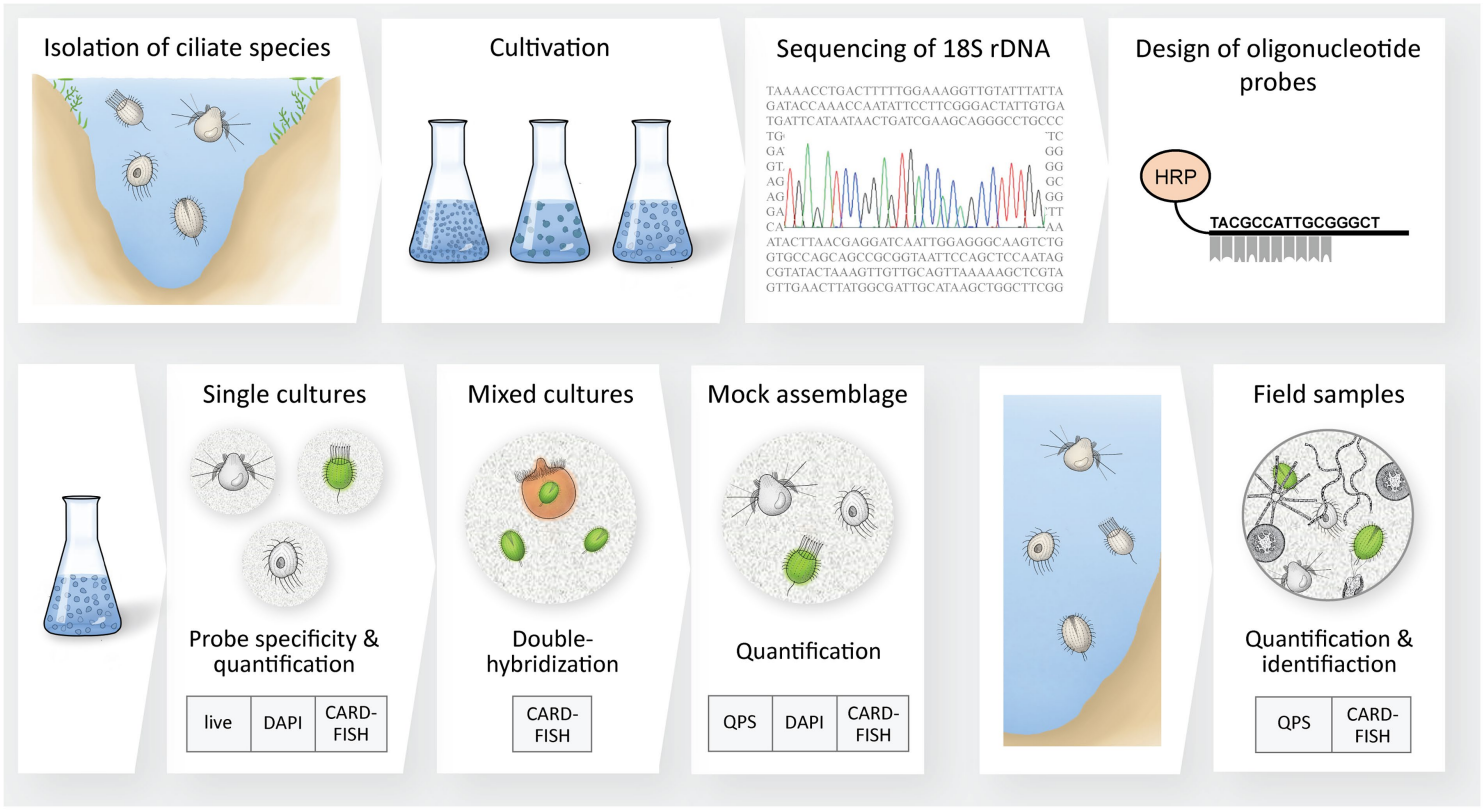
Overview of the workflow used to elaborate a protocol for the identification and quantification of planktonic freshwater ciliates *via* CARD-FISH. Nine ciliates species were collected and cultivated before their 18S rDNA was sequenced. Newly designed oligonucleotide probes for CARD-FISH were designed and first tested on cultures (single and mixed cultures, mock assemblage) and thereafter on ciliates from lake water samples. We tested the specificity of all probes and proved the quantitative properties of CARD-FISH by comparisons between live cell counts, QPS and DAPI preparations.

All nine selected ciliate species originate from freshwater habitats (Figure 1; Table 1). Species identification was done with living and protargol-stained (QPS, Skibbe, 1994; Pfister et al., 1999) specimens using different identification keys (Foissner et al., 1994, 1999) and recently published work (Frantal et al., 2022; Sonntag et al., 2022). Taxonomic classification was updated according to Lynn (2008). Ciliates were kept in culture flasks (25 cm^2^; Techno Plastic Products, Switzerland) with pre-filtered mineral water (Volvic, France), at 18°C and under a 12:12 h day:night cycle with an insolation of 1.05 W m^−2^. The food source of *Cinetochilum margaritaceum* consisted of bacteria grown in a suspension of autoclaved wheat grains. All other species were fed with the algae *Cryptomonas* sp. (strain SAG 26.80, Culture Collection of Algae, University of Göttingen, Germany). The ciliate *Monodinium chlorelligerum* received *Urotricha cf. pseudofurcata* as an additional food source.

**Table 1:** Information regarding the nine examined ciliate species on which the CARD-FISH method was tested and modified. Detailed information on oligonucleotide probes can be found in Supplementary Table S2. Species where an unambiguous identification was not possible are labeled with *cf.* (Latin for confer/conferatur).

Taxonomy	Isolated from	Food	Accession number GenBank	Card-FISH probe	
				Species-specific	Genus-specific
Litostomatea	Lake Zurich	*Cryptomonas* sp.	OU230608	Ask-193	-
*Askenasia cf. volvox*					
Litostomatea	Lake Zurich	*Cryptomonas* sp. & *U. cf. pseudofurcata*	OU230633	MonoZH-179	Mono-all-826
*Monodinium chlorelligerum*					
Oligohymenophorea	Lake Zurich	Bacteria grown on wheat grains	OU230529	Cin-1237	-
*Cinetochilum margaritaceum*					
Prostomatea	Lake Zurich	*Cryptomonas* sp.	OU230630	Bal-651	-
*Balanion planctonicum*					
Prostomatea	Lake Mondsee	*Cryptomonas* sp.	MW077193^a^	Uro2-1440	Uro-all-651
*Urotricha cf. castalia*^a^					
Prostomatea	Lake Faselfad	*Cryptomonas* sp.	ON176661^b^	Uro4-1436	Uro-all-651
*Urotricha cf. nais*^b^					
Prostomatea	Lake Zurich	*Cryptomonas* sp.	OU230631	Uro5-403	Uro-all-651
*Urotricha cf. pseudofurcata*^a^					
Spirotrichea	Lake Zurich	*Cryptomonas* sp.	OU230632	HalZH-1133	Hal-all-1362
*Halteria cf. bifurcata*					
Spirotrichea		*Cryptomonas* sp.	OU230607	-	Hal-all-1362
*Halteria grandinella*	Cultivation tanks ofaquatic plant *U.reflexa*^c^				

a Detailed description in Frantal et al. (2022).

b Detailed description in Sonntag et al. (2022).

c Cultivated by Lubomír Adamec, Institute of Botany CAS, Section of Plant Ecology, Třeboň 379 82, Czech Republic.

### Amplification and sequencing of rRNA genes

The 18S rRNA genes of *C. margaritaceum*, *Urotricha cf. castalia*, and *Urotricha cf. nais* were amplified and sequenced as described in Pitsch et al. (2019) and Frantal et al. (2022). For the other six ciliate species (Table 1) we proceeded as follows: Up to 50 cells were washed in several drops of mineral water and starved for 12 to 24 h. Single or multiple cells (max. 10) were picked using a drawn glass micropipette and stored in maximum 20 μl mineral water at −20°C. In a next step, the aliquots were thawed to rupture the cells. 18S rDNA was amplified by PCR without prior DNA extraction applying the following protocol: First denaturation at 95°C for 30 s followed by 35 cycles, each consisting of 30 s at 95°C, 30 s at 55°C, 2 min and 30 s at 72°C, followed by a final elongation for 5 min at 72°C using the DreamTaq™ Green PCR Master Mix (2×, Thermo Fisher Scientific, United States) and different primers targeting the 18S rDNA (Supplemenatry Table S1). Successfully amplified products were purified with Agencourt AMPure XP PCR Purification Kit (Beckman Coulter, United States) and Sanger sequenced using the BrilliantDye® Terminator Cycle Sequencing kit v3.1 (NimaGen, Netherlands) on an ABI 3730 Genetic Analyzer (Applied Biosystems, Thermo Fisher Scientific, United States). Sequences were finally assembled using the software DNA Baser v2.91.5 (Heracle BioSoft SRL) and Geneious® 9.1.7 (Biomatters Ldt).

### Phylogenetic analysis and design of ciliate probes for CARD-FISH

The 18S rDNA of the nine studied ciliate species served as a basis for the design of species- and genera- specific oligonucleotide probes for CARD-FISH using the program ARB (Ludwig et al., 2004). Initial sequence alignments were conducted with SINA aligner (Pruesse et al., 2012) and imported to ARB using SILVA database SSURef_NR99_138.1 (Pruesse et al., 2007). After manual curation of alignments, maximum likelihood trees (GTR-GAMMA, 1000 bootstraps, Stamatakis et al., 2005) of the ciliate 18S rDNA sequences, including their closest relatives, were generated (Supplementary Figures S1,S2). Based on these phylogenetic trees, oligonucleotide probes as well as corresponding competitor oligonucleotides were designed with the ARB tools probe_design and probe_check and analyzed with mathFISH (Yilmaz et al., 2011). Among other outputs, the software suggests the range of formamide concentrations which should be tested to assess probe specificity. In addition to eight species- and three genera-specific oligonucleotide probes (*Halteria, Monodinium, Urotricha*), a general eukaryote-specific probe was designed by modifying the original probe (Lim et al., 1993; Table 1; Supplementary Table S2). All probes were 5′ labeled with horseradish peroxidase (HRP; Sekar et al., 2003).

### CARD-FISH procedure

For testing probe specificity, determining optimal formamide concentrations, and finding optimal quantification strategies, we performed CARD-FISH on single ciliate cultures, mock assemblages and lake water samples. The general procedure followed, with some adaptations, the protocol by Piwosz et al. (2021). A ready-to use as well as a detailed protocol and information about filters, solutions and buffers are provided in Supplementary Figure S3; Supplementary File S1, and Supplementary Table S3. First, cultures or lake water samples were preserved with pre-filtered formaldehyde [~2% final concentration (f.c.)]. Fixed samples were stored for at least 1 h at 4°C in the dark, subsequently gently filtered (< 50 mbar) onto polycarbonate membrane filters (1.2 or 3 μm pore size, Merck Millipore, Germany), and washed twice with 10 ml 1x phosphate buffered saline (PBS). To prevent detachment of cells from the filters, they were embedded in 0.1% agarose and dried on parafilm at 37°C for 30 min. Enzymatic permeabilization of the cell wall was not needed for the hybridization of ciliates. Filters were incubated in 0.01 M HCl for 20 min at room temperature to inactivate endogenous peroxidases. Subsequently, samples were washed in PBS, deionized sterile water and dehydrated in ethanol (1 min each). After cutting filters into six or eight pieces, the individual sections were transferred to an Eppendorf tube with a mixture containing hybridization buffer, the specific oligonucleotide probe (50 μg ml^−1^) and, if required, the corresponding competitors (50 μg ml^−1^) in a ratio of 150:1:1 and incubated at 35°C for 3 h. Afterwards, filter sections were transferred into 50 ml of pre-warmed washing buffer and incubated for 30 min at 37°C. To equilibrate the probe-delivered HRP, the filter sections were incubated in PBS/Triton X-100 solution (PBST) for 45 min at 37°C. Sections were then dabbed onto blotting paper, placed into the tyramide solution mixture (amplification buffer, H_2_O_2_, fluorochrome-labeled tyramides) and incubated in the dark for 30 min at 37°C. After this tyramide signal amplification step, sections were again dabbed onto blotting paper to remove excess liquid and incubated in PBST for 15 min at room temperature in the dark. They were subsequently washed for approximately 1 min in deionized sterile water and EtOH. The air-dried sections were finally mounted on glass slides, embedded and counterstained with a custom mix of mountant amended with 4′, 6′-diamidino-2-phenylindole (DAPI-Mix, Supplementary Table S3) and stored at −20°C until further processing.

### DAPI and QPS preparations

For some experiments, filters only stained with DAPI were prepared in addition to CARD-FISH preparations to evaluate the effect of fixatives on the cell integrity of ciliates (Figure 1). A defined amount of fixed ciliate culture was first filtered onto polycarbonate filters (PCTE membrane disk black, 1 μm pore size, GVS, Italy), supported by cellulose nitrate filters (5 μm pore size, Sartorius, Germany) and stained with DAPI (Porter and Feig, 1980). Subsequently, the filters were placed onto glass slides, embedded with immersion oil (Typ A, Cargille, United States) and stored at −20°C until further processing.

QPS was carried out in two additional experiments for an accurate reference quantification of ciliates (Figure 1). Ciliate cultures or water samples were preserved with Bouin’s solution (5% f.c., Skibbe, 1994) and stored at room temperature. Within 3 to 6 weeks, the preserved samples were filtered through 0.8 μm pore sized cellulose nitrate filters with counting grids (Sartorius, Germany) and embedded in agarose (Agar Noble Difco™, Becton Dickinson, United States). Silver impregnation was performed following the protocol of Skibbe (1994) with slight modifications after Pfister et al. (1999). After the staining procedure, silver impregnated ciliates on filters were embedded in Canada balsam (Merck Millipore, Germany) to produce permanent slides.

### Microscopy and image processing

CARD-FISH, DAPI and QPS preparations were evaluated on an Axio Imager.M1 microscope (Zeiss, Germany) at 400–630× magnification, equipped with an HBO 100 mercury lamp and different filter sets for epifluorescence (Supplementary Table S4). Fluorescently labeled ciliates were visualized with the Zeiss filter sets 62 HE and 43 for the detection of the hybridization signals, depending on the fluorochrome (fluorescein isothiocyanate (FITC) or Alexa Fluor 546). The Zeiss filter set 01 was used for UV excitation, i.e., to visualize signals after DAPI staining. Additionally, we tested the Zeiss filter set 10 for the detection of hybridized cells. Recording of microphotographs and measurements were performed with the software AxioVision Rel. 4.8 (Zeiss). Image arrangements and minor contrast enhancements were carried out with the software Adobe Illustrator CS5 (vers.15.0.0) and Adobe Photoshop CS5 (vers. 12.0 ×64), respectively.

### Tests with cultures

#### Optimal formamide concentration and probe specificity

CARD-FISH was first performed separately with each ciliate culture and the respective newly designed species- and/or genus-specific probe to establish the optimal formamide concentration in the hybridization buffer. Formamide concentrations between 40% and 90% were tested. To verify the specificity of the 12 oligonucleotide probes, CARD-FISH was conducted with all probes against each single culture. The specificity of the probes Bal-651, Ask-193 and Cin-1237 were additionally tested with a mock assemblage consisting of *Askenasia cf. volvox*., *Balanion planctonicum*, and *C. margaritaceum*.

#### Double hybridization—*Monodinium chlorelligerum* and *Urotricha cf. pseudofurcata*

The CARD-FISH procedure for double hybridizations followed the general protocol described above with slight adaptations according to Supplementary File S3 in Piwosz et al. (2021). A starving culture of *M. chlorelligerum* was fed with *U. cf. pseudofurcata* and fixed with formaldehyde after 24 h. Filter sections were first hybridized with the *U. cf. pseudofurcata-*specific probe (Uro5-403) using FITC labeled tyramides for signal amplification. After the last washing step, the protocol was repeated with the same filter sections. The HRP from the previous hybridization was inactivated by incubating the filters in HCl for 10 min. A second hybridization was then performed with the *M. chlorelligerum*-specific probe (MonoZH-179), and signal amplification was conducted with Alexa Fluor 546 labeled tyramides.

#### Live versus DAPI and CARD-FISH counts

To evaluate the quantitative nature of CARD-FISH (Figure 1), an experiment was conducted with all ciliate cultures and their corresponding probes, except for *Halteria cf. bifurcata,* because this culture had been lost by that time. As a first step, ciliates were counted alive in small drops (10–30 μl) with a stereomicroscope (Zeiss Discovery.V8) aiming a total number of at least 250 cells. The entire culture was subsequently fixed with formaldehyde before one CARD-FISH and triplicate DAPI filters were prepared. An adequate volume from each preserved culture was filtrated to obtain at least 1,000 cells per filter. Hybridized cells on three filter sections (1/8 or 1/6 of whole filter) were counted on CARD-FISH preparations, as well as a minimum of 250 cells on each of the triplicate DAPI filters. The number of hybridized/stained ciliates on filters were compared to the respective live counts. Only 156 cells of *M. chlorelligerum* were counted live, because this culture did not grow to higher densities. Therefore, the subsequent CARD-FISH preparation and evaluation of these ciliates was performed on a small filter (TSTP, 25 mm Ø, Supplementary Table S3) cut into two pieces, and only duplicate DAPI filters of *M. chlorelligerum* were prepared and evaluated. In addition, a large proportion of *M. chlorelligerum* cells were lost during the CARD-FISH procedure. Thus, apart from testing alternative fixatives (as outlined below for *Halteria grandinella)*, we also tested the embedding of these ciliates in agarose with a higher concentration (0.3%) prior to hybridization. *H. grandinella* seemed to be very fragile, and many cells were observed to rupture during filtration and hybridization. Therefore, higher concentrations of formaldehyde (3.5% f.c.), as well as additional fixatives were tested before CARD-FISH and DAPI filters were prepared, namely mixtures of Lugol’s solution (1% f.c.) and formaldehyde (~2% f.c.; Piwosz et al., 2021), and ethanol (~30% f.c.) and formaldehyde (4.5% f.c; Foissner, 2014).

#### Quantification of ciliates in a mock assemblage

A mock assemblage comprising *B. planctonicum*, *C. margaritaceum* and *A*. *cf. volvox* was used to test the quantification by CARD-FISH as compared to DAPI and QPS preparations (Figure 1). Each species was first counted alive as described above. In order to obtain approximately equal proportions (500 cells) per species per filter (i.e., 1,500 cells in total), corresponding volumes from each culture were mixed and then fixed with either pre-filtered formaldehyde (~2% f.c.) for DAPI and CARD-FISH, or with Bouin’s solution (5% f.c.; Skibbe, 1994) for QPS. To evaluate the abundance of DAPI stained cells in the mock assemblage, triplicate filters were prepared and a minimum of 450 cells were identified and counted for each replicate. For CARD-FISH, five filters (TSTP, 25 mm Ø, Supplementary Table S3) were prepared and cut into four uniform sections. One quarter was then hybridized with the *A. cf. volvox*- (Ask-193), one with the *B. planctonicum-* (Bal-651) and one with the *C. margaritaceum*- (Cin-1237) specific probe. At least 600 hybridized cells were counted per species. Furthermore, four silver-impregnated filters were produced by QPS and at least 400 ciliate cells were counted per filter. In addition, CARD-FISH was performed on the mock assemblage after a 1:20 dilution with lake water. This was done to determine whether signals of hybridized ciliates were sufficiently strong to distinguish them from a background of other (biotic and abiotic) fluorescent particles. Five CARD-FISH filters were prepared, hybridized and evaluated as described above. The water for the dilution was collected from Lake Zurich (Switzerland) on 8 January, 2020 (see next paragraph), preserved with formaldehyde (~2% f.c.) and stored at 4°C in the dark. During this time of the year, the three studied ciliates are usually present in very low numbers in the lake (Pitsch et al., 2019 and unpubl. observations).

### CARD-FISH with lake water samples

Water samples from Lake Zurich (Switzerland) were collected weekly at a depth of 1 m to 8 m with an integrating sampler (Uwitec, Austria) during spring 2020. All samples were transported to the laboratory within 1 h, where 2 × 200 ml were fixed with formaldehyde (~2% f.c.) for later CARD-FISH. In addition, 300 ml of lake water was preserved with Bouin’s solution (5% f.c.). For QPS, 100 ml of preserved water samples were filtered and processed as described above. A minimum of 400 ciliates were counted and identified using the taxonomic key published by (Foissner et al., 1999). For CARD-FISH, 2 × 200 ml of fixed samples were filtrated (TSTP, 47 mm Ø, Supplementary Table S3), embedded in agar and stored at −20°C until further processing. Hybridization was done with oligonucleotide probes specific for the species *C. margaritaceum* (Cin-1237), *B. planctonicum* (Bal-651) and *U. cf. pseudofurcata* (Uro5-403) as well as for the genera *Urotricha* (Uro-all-651) and *Halteria* (Hal-all-1362). These probes were selected because the respective ciliate morphotypes were previously found to be abundant in Lake Zurich (Posch et al., 2015; Pitsch et al., 2019). Hybridized cells were counted on three to six filter sections (1/6) and their numbers were compared with the corresponding counts from QPS filters.

### Statistical analyses

All statistical analyses were carried out with the R software (version 4.1.1). Parametric and non-parametric statistics were performed to evaluate if CARD-FISH is suitable for ciliate quantification. For the first experiment, live cell counts from eight ciliate cultures were compared to counts from DAPI and CARD-FISH treatments by one-way ANOVA followed by Tukey’HSD posthoc tests or Kruskal-Wallis tests followed by Dunn’s tests (package “dunn.test”) if the assumptions of ANOVA were not satisfied (e.g., for *A. cf. volvox* and *H. grandinella*). False discovery rate of multiple testing was corrected using the Benjamini-Hochberg method (Benjamini and Hochberg, 1995). A two-way ANOVA was used to assess the effect of the method (QPS, DAPI and CARD-FISH) and the ciliate species on the cell counts in the mock assemblage. In addition, CARD-FISH counts of the mock assemblages were compared with counts from the mock assemblage mixed with lake water. For this purpose, counts from replicates were pooled and a Chi-square goodness of fit test was performed (package “stats”). No replicate was available for QPS of lake water samples. Thus, to compare QPS counts of *C. margaritaceum*, *Urotricha* spp. ≤35 μm and *Halteria* spp. from lake water samples with average cell-counts by CARD-FISH, the ratios between cell counts of the two methods were calculated for each sampling date and species/genus. Next, the ratios for each ciliate species/genus were compared to the expected proportion of 1 using one sample t-tests. False discovery rate of multiple testing was corrected using the Benjamini–Hochberg method (Benjamini and Hochberg, 1995).

## Results

### Probe specificity and formamide concentrations

**Figure 2:**
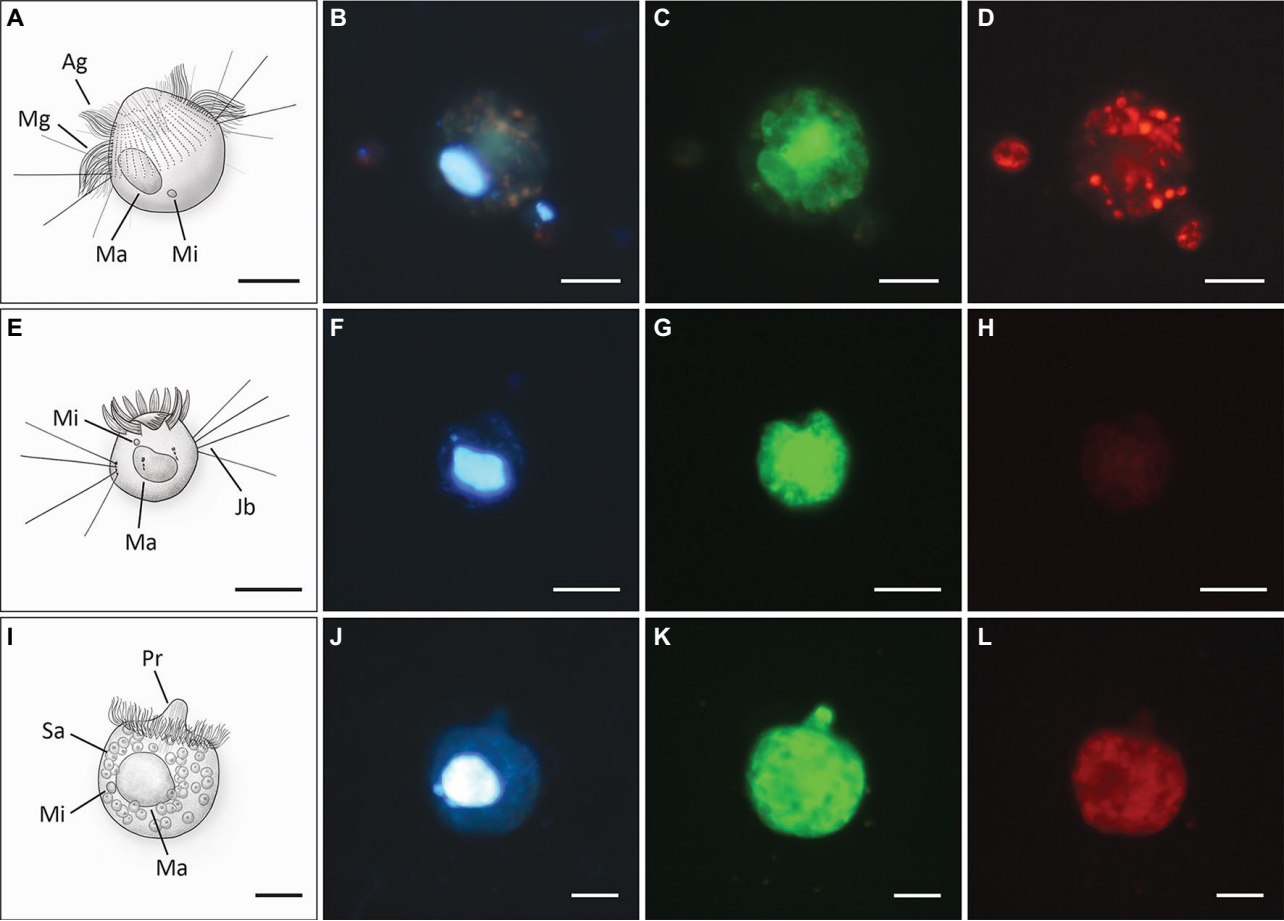
Drawings (first column) and associated photographs of CARD-FISH of *A*. *cf. volvox* **(A–D)**, *H. grandinella* **(E–H)** and *M. chlorelligerum* **(I–L)**. Photographs in each row show the same cell and were taken with an epifluorescence microscope using different optical filters to visualize macro- and micronucleus stained with DAPI **(B, F, J)**, cells hybridized with specific probes, i.e., *A*. *cf. volvox* with probe Ask-193 **(C)**, *H. grandinella* with probe Hal-all-1362 **(G)**, *M. chlorelligerum* with probe Mono-all-826 **(K)**, and autofluorescence of ingested *Cryptomonas* sp. **(D)**, algal endosymbionts of *M. chlorelligerum* **(L)**. (Ag) anterior girdle of ciliary tufts, (Jb) jumping bristles, (Ma) macronucleus, (Mg) middle girdle of ciliary tufts, (Mi) micronucleus, (Pr) proboscis, (Sa) symbiotic algae. Scale bars = 20 μm.

All probes listed in Table 1 were species- or genus- specific, and most hybridized ciliates emitted a strong fluorescence signal (Table 2; Figure 2). Likewise, the modified general eukaryotic probe EUK1209-mod hybridized with all examined ciliates. Only the probe Uro-all-651 initially gave a false positive signal as it also hybridized *B. planctonicum* cells. This could be prevented by including the oligonucleotide Bal-651 as an additional competitor (Table 2; Supplementary Table S2). The probe Mono-all-826 was tested with *M. chlorelligerum* only, and its specificity for the genus *Monodinium* has not yet been demonstrated. The optimal specificity of newly designed probes were reached with formamide concentrations between 50 and 70% in hybridization buffer (Supplementary Table S2). Increasing either hybridization temperature or time did not improve the signal quality. Signals of hybridized cells were very pronounced even at low tyramide solution concentrations (2 μl). Increased concentrations usually resulted in higher background signals.

**Table 2:** Specificity of CARD-FISH probes. All nine studied ciliate species (first column) versus all 12 tested oligonucleotide probes (first row). Positive signals of hybridized cells are highlighted in green and false positive signals are highlighted in red. Signal intensity: +++ strong, ++ moderate, + weak. Grey: - no signal.

	Ask-193	Mono-ZH-179	Mono-all-826	Cin-1237	Bal-651	Uro2-1440	Uro4-1436	Uro5-403	Uro-all-651		Hal-ZH-1133	Hal-all-1362	EUK1209-mod
*A. cf. volvox*	**+++**	**−**	**−**	**−**	**−**	**−**	**−**	**−**	**−**		**−**	**−**	**+++**
*M. chlorelligerum*	**−**	**+**	**++**	**−**	**−**	**−**	**−**	**−**	**−**		**−**	**−**	**+**
*C. margaritaceum*	**−**	**−**	**−**	**+++**	**−**	**−**	**−**	**−**	**−**		**−**	**−**	**+++**
*B. planctonicum*	**−**	**−**	**−**	**−**	**+++**	**−**	**−**	**−**	**+**	**−**	**−**	**−**	**+++**
*U. cf. castalia*	**−**	**−**	**−**	**−**	**−**	**+++**	**−**	**−**	**+++**		**−**	**−**	**+++**
*U. cf. nais*	**−**	**−**	**−**	**−**	**−**	**−**	**++**	**−**	**+++**		**−**	**−**	**+++**
*U. cf. pseudofurcata*	**−**	**−**	**−**	**−**	**−**	**−**	**−**	**+++**	**+++**		**−**	**−**	**+++**
*H. cf. bifurcata*	**−**	**−**	**−**	**−**	**−**	**−**	**−**	**−**	**−**		**+**	**++**	**+++**
*H. grandinella*	**−**	**−**	**−**	**−**	**−**	**−**	**−**	**−**	**−**		**−**	**++**	**+++**

### Double hybridization

**Figure 3:**
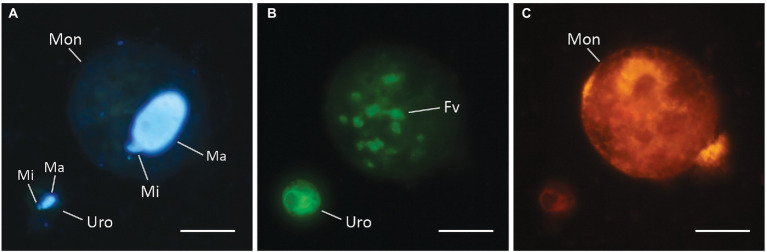
CARD-FISH photographs of double hybridization of the prey *U. cf. pseudofurcata* and its predator *M. chlorelligerum*. Photographs show the same area on the filter and were taken using different optical filters. DAPI-staining visualizing macro- and micronucleus of both ciliate species **(A)**, *U. cf. pseudofurcata* hybridized with Uro5-403 **(B)**, and *M. chlorelligerum* hybridized with MonoZH-179 **(C)**. In the left bottom corner of **(B)** an intact *U. cf. pseudofurcata* cell and inside the predator several ingested and largely digested cells and cell remains are shown. Different colors of hybridized cells derive from different dyes used for CARD-FISH. (Ma) Macronucleus, (Mi) micronucleus, (Mon) *M. chlorelligerum*, (Fv) food vacuoles with cell remains of *U. cf. pseudofurcata*, (Uro) *U. cf. pseudofurcata*. Scale bars = 20 μm.

Double hybridization with the probe Uro5-403 for *U. cf. pseudofurcata* (prey) and MonoZH-179 for *M. chlorelligerum* (predator) resulted in a clear signal, and the two species could easily be distinguished (Figure 3). Strikingly, engulfed and partially digested *Urotricha* still maintained a strong signal inside *M. chlorelligerum* cells (Figure 3B).

### Quantification

**Figure 4:**
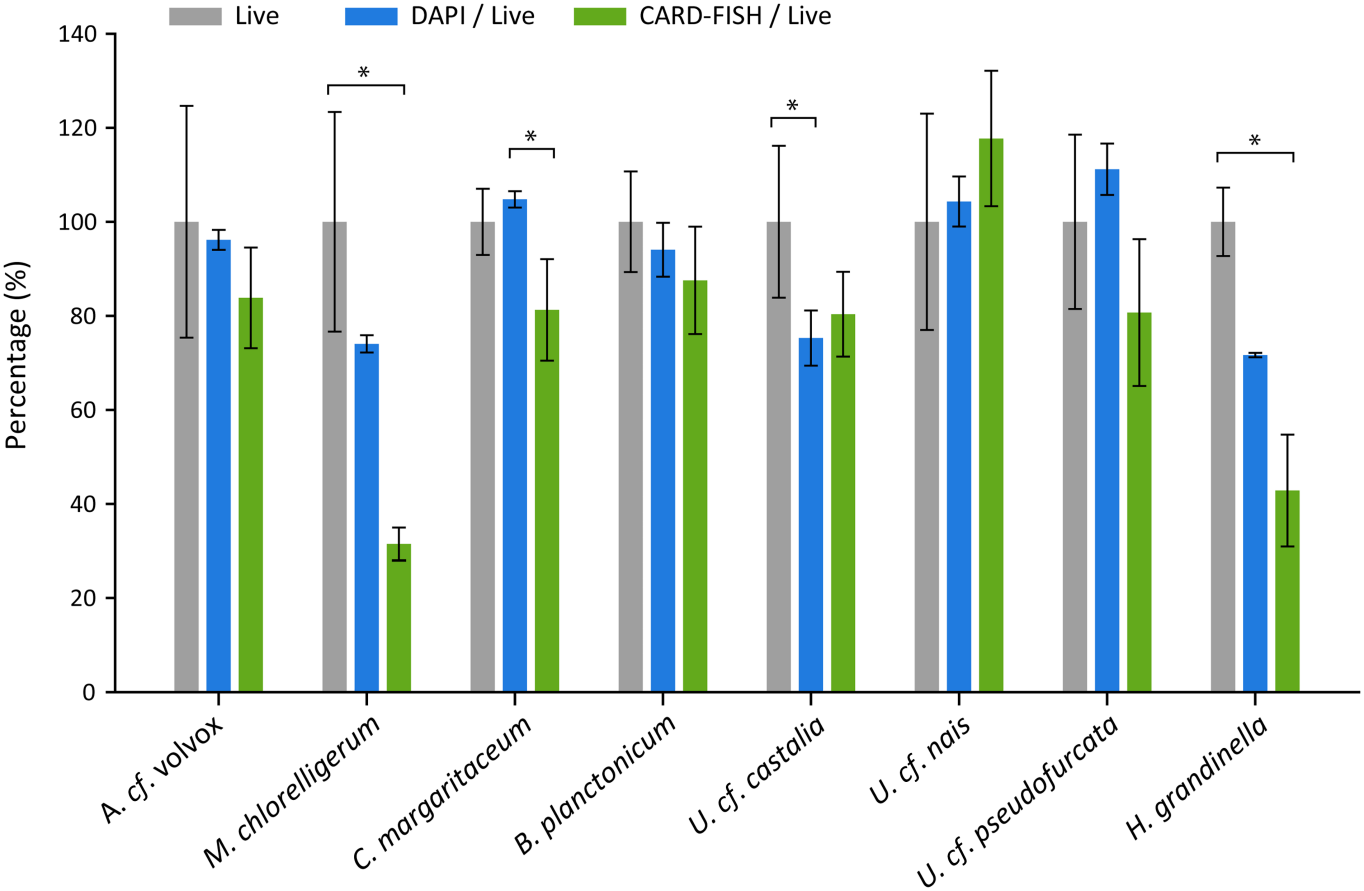
Live counts (grey, set to 100%) compared to those of DAPI (blue) and CARD-FISH (green) for eight ciliate species. *H. grandinella* was hybridized with its genus-specific probe, all other ciliates with their species-specific probe (Tables 1,2). Error bars show standard deviations and stars (*) indicate significant differences between methods (*p* < 0.05). Absolute cell abundances of the same treatments are shown in Supplementary Figure S4.

**Figure 5:**
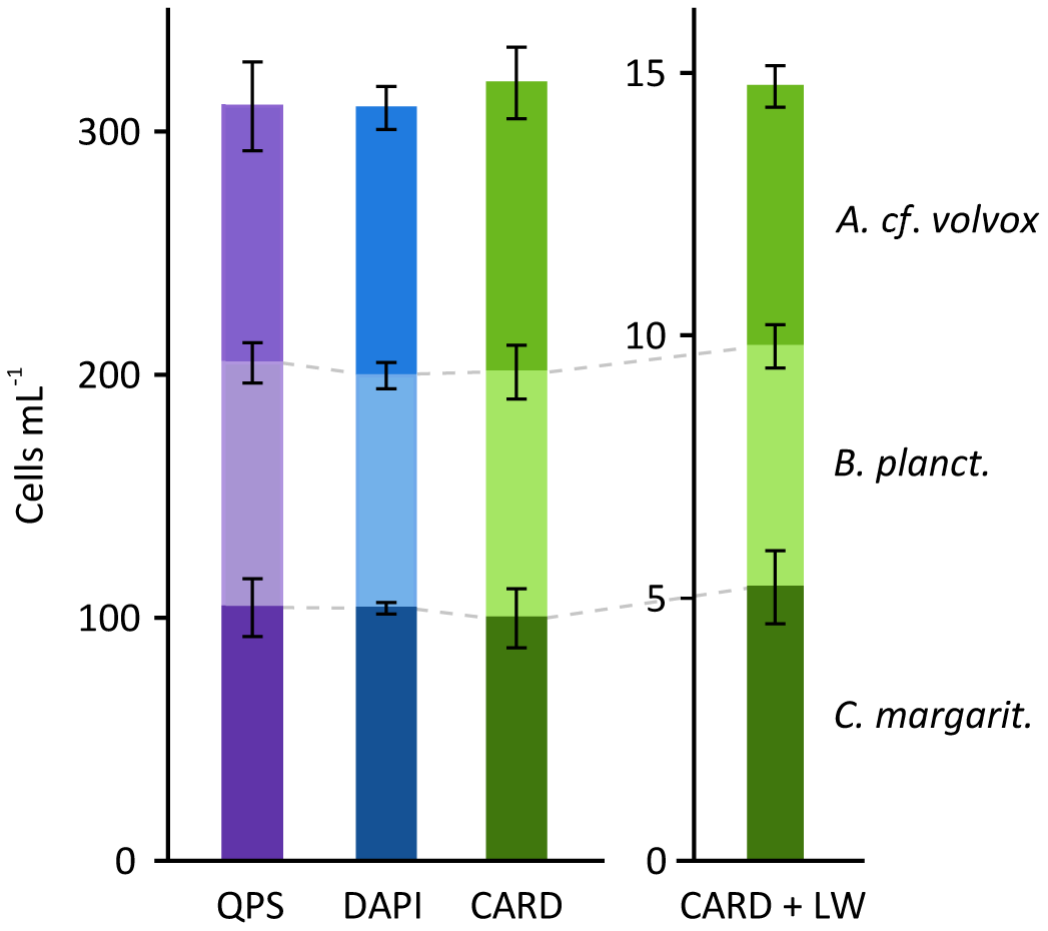
Quantification of cell abundances in a mock assemblage (*A*. *cf. volvox*, *C. margaritaceum*, *B. planctonicum*) from QPS (violet), DAPI-stained (blue) and hybridized cells (CARD-FISH, green). Each species was hybridized with its species-specific probe (Tables 1,2). CARD-FISH was additionally performed with the same mock assemblage mixed with lake water (CARD + LW). Error bars show standard deviations.

**Figure 6:**
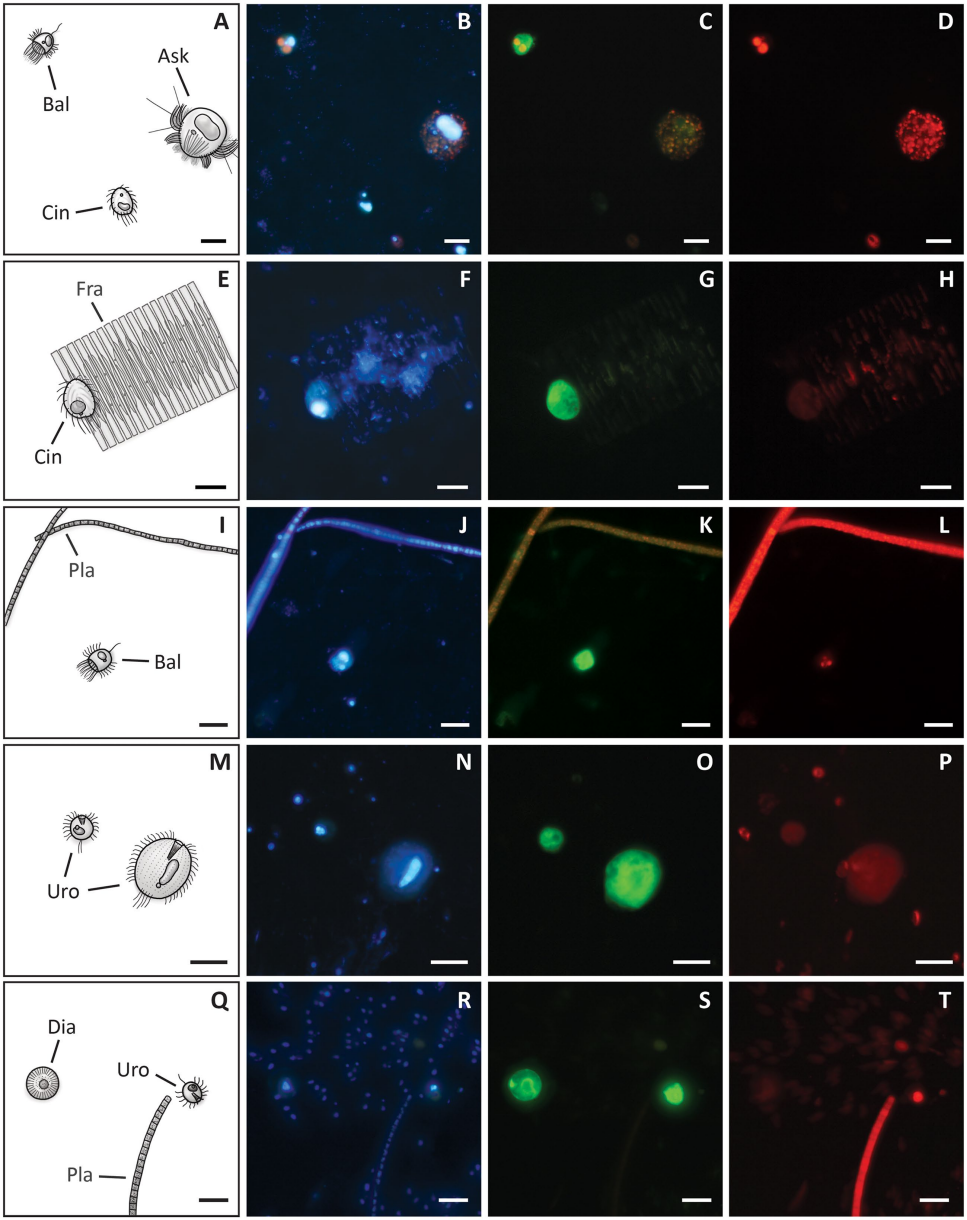
Drawings (first column) and associated photographs of CARD-FISH of the mock assemblage and water samples from Lake Zurich. Photographs in each row show the same area on the filter and were taken using different optical filters visualizing DAPI-staining of nuclei (second column), hybridized cells (third column) and chlorophyll *a* autofluorescence (fourth column). CARD-FISH was performed with the mock assemblage and *B. planctonicum*-probe Bal-651 **(A–D)**, the mock assemblage mixed with lake water and *C. margaritaceum*-probe Cin-1237 **(E–H)**, lake water and *B. planctonicum*-probe Bal-651 **(I–L)**, and lake water with the *Urotricha* genus-specific probe Uro-all-651 **(M–T)**. Note ingested algae within *A*. *cf. volvox* **(D)**, *B. planctonicum* **(D, L)** and *Urotricha* spp. **(P, T)**, and the false positive signal of centric diatoms **(S)**. The drawings present some organisms found on the CARD-FISH preparations: (Ask) *A*. *cf. volvox*, (Bal) *B. planctonicum*, (Cin) *C. margaritaceum*, (Dia) centric diatom, (Frag) *Fragilaria* sp., (Pla) *Planktothrix rubescens*, (Uro) *Urotricha* sp. Scale bars = 20 μm.

The experiments showed that CARD-FISH was an appropriate method for quantifying various ciliate species. Tests comparing counts of living, DAPI and CARD-FISH-stained cells resulted in highly congruent numbers for *A. cf. volvox*, *B. planctonicum*, *U. cf. pseudofurcata* and *U. cf. nais*, while there were moderate differences for other species (Figure 4; Supplementary Figure S4; Supplementary Tables S5,S6). Numbers of *H. grandinella* cells differed, although not statistically significant, among methods (Kruskal–Wallis, *H* = 8.067, df = 2, *p* = 0.069), in particular between live and CARD-FISH cell counts (Dunn’s test, *p* = 0.007): The cell numbers detected after CARD-FISH accounted only for 42% of live cell counts. Destroyed cells were repeatedly observed on filters from both CARD-FISH and DAPI treatments. Different fixatives did not enhance the stability of *H. grandinella* cells (Supplementary Figure S5;Supplementary Tables S7,S8), even though all fixation methods resulted in a clear signal of hybridized cells. After CARD-FISH, the highest numbers were found with cells fixed with formaldehyde (~2 and 3.5% f.c.), accounting for maximal ~60% of live cells counts. A one-way ANOVA also indicated discrepancies between the methods for *M. chlorelligerum* (*F*_(2, 5)_ = 7.081, p = 0.069). The number of CARD-FISH-stained cells were by approximately two thirds lower than those of live counting (Tukey’s HSD, *p* = 0.029). Using a mix of Lugol’s solution and formaldehyde as an alternative fixative or increasing the agar concentration for CARD-FISH did not improve the outcome (Supplementary Figure S5; Supplementary Tables S7,S8). Variation among methods, albeit not statistically significant, was also evident for *C. margaritaceum* (ANOVA, *F*_(2, 7)_ = 5.954, p = 0.069) and *U. cf. castalia* (*F*_(2, 15)_ = 6.541, p = 0.069). However, CARD-FISH and live cell counts were similar for both, *U. cf. castalia* (Tukey’s HSD, *p* = 0.11) and *C. margaritaceum* (Tukey’s HSD, *p* = 0.07). The analysis of the mock assemblage, comprising the species *A*. *cf. volvox*, *C. margaritaceum* and *B. planctonicum,* further confirmed the quantitative accuracy of CARD-FISH (Figure 5). The method had no influence on cell numbers (two-way ANOVA, *F* = 0.452, df = 2, *p* = 0.66) and this was not affected by ciliate species (*F*_(2,4)_ = 0.975, *p* = 0.43). Furthermore, diluting the mock assemblage with lake water did not affect the proportion of cell counts (Chi-square goodness-of-fit, *χ*^2^ = 6, df = 4, *p* = 0.199), and the three species were easily discriminated from other protists in the lake water sample by their fluorescent signal (Figures 6A–H).

### Lake water samples

CARD-FISH preparations with lake water samples using specific ciliate probes resulted in unambiguous staining, except for one species. The studied ciliates were clearly distinguishable from other microorganisms found in Lake Zurich during spring 2020 (Figures 6I–T). However, particularly small centric diatoms (Figure 6S) and, to a lesser extent, plates of dinoflagellates and structures of larger zooplankton (e.g., rotifers, copepods, daphnids) emitted a similar, strong fluorescence signal. Such false positive signals, likely due to unspecific binding of tyramides, was observed with all probes used in this study.

**Figure 7:**
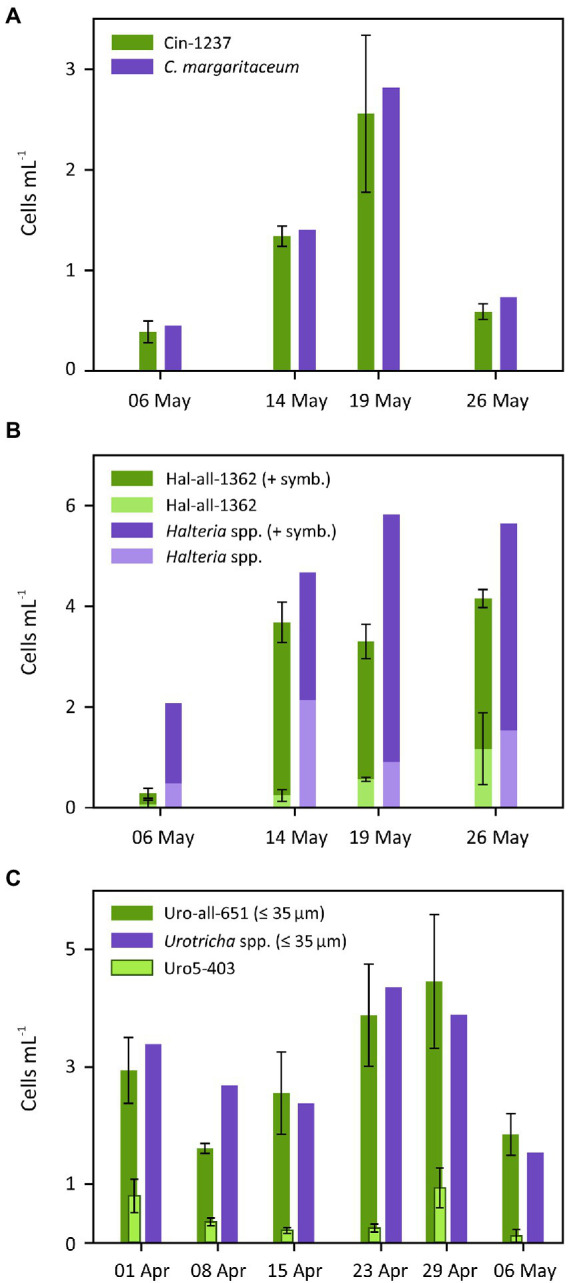
Comparison of counts obtained from CARD-FISH (green) and QPS (violet) preparations from water samples collected in Lake Zurich during spring 2020. Cells hybridized with *C. margaritaceum*-probe Cin-1237 (CARD-FISH) compared to protargol-stained *C. margaritaceum* (QPS, **A**), cells hybridized with genus-specific *Halteria*-probe Hal-all-1362 compared to *Halteria* spp. from QPS, quantified with and without algal symbionts (dark and light color, respectively, **B**), and hybridization with Uro-all-651-probe for *Urotricha* spp. compared with *Urotricha* spp. from QPS **(C)**. Lake water samples were additionally hybridized with Uro5-403 to look for *U. cf. pseudofurcata* (light green bars in **C**). Error bars show standard deviations. Note that no replicate was available for QPS preparations.

With the exception of *B. planctonicum,* QPS and CARD-FISH with lake water samples resulted in comparable cell abundances, with variation depending on the species/genus and sampling date (Figure 7). *B. planctonicum* was found to be abundant (> 4,000 cell L^−1^) during spring and early summer 2020, as assessed by QPS staining. In contrast, almost no *B. planctonicum* were found on CARD-FISH filters, i.e., hardly any cells were hybridized with probe Bal-651 (Figures 6I–L). Adjustments such as lowering formamide concentrations in the hybridization buffer (down to 30%), increasing the probe/competitor concentration or creating a new probe targeting another region did not improve the outcome (see discussion for further details). The best match between QPS and CARD-FISH was found for *C. margaritaceum.* Cell numbers hybridized with probe Cin-1237 or identified as *C. margaritaceum* on QPS filters were similar in all four lake water samples (Figure 7A). The ratio of cell counts by the two methods did not significantly differ from 1 (mean ratio = 0.88; *t*-test, *t* = 3.64, df = 3, *p* = 0.09). Using the genus specific probe for *Halteria* spp., Hal-all-1362, cells with and without algal endosymbionts were detected. The same was true for cells stained with QPS. A discrepancy between methods was evident when comparing numbers of *Halteria* cells on separate days (Figure 7B). However, when considering all days together, the ratios of QPS-stained to hybridized cells numbers did not differ significantly from 1, regardless whether the total number of cells (mean ratio = 0.55; *t*-test, *t* = 3, df = 3, *p* = 0.10), the number of cells with (mean ratio = 0.69; *t*-test, *t* = 1.21, df = 3, *p* = 0.39) or without symbionts (mean ratio = 0.4; *t*-test, *t* = 3.57, df = 3, *p* = 0.09) were taken into account. However, morphological details observed in QPS samples (i.e., the shape of jumping bristle complexes) suggested that endosymbiont-bearing cells belonged to the species *Pelagohalteria viridis* rather than to the genus *Halteria*. Due to hardly distinguishable morphological features after QPS, *Urotricha* spp. were assigned to a specific size range rather than to a particular species. For a comparison with CARD-FISH, i.e., hybridization with the Uro-all-651 probe, only *Urotricha* spp. ≤ 35 μm were considered, as this size class also included the largest studied species, *U. cf. castalia*. Comparing counts of *Urotricha* spp. from QPS and CARD-FISH preparations showed good agreement, although some variation was observed between days (Figure 7C). Summarizing all dates, the ratio of cell counts between the two methods did not differ significantly from 1 (mean ratio = 0.96; *t*-test, *t* = 0.40, df = 5, *p* = 0.70). Interestingly, hybridization with the probe Uro5-403, specific for *U. pseudofurcata* (Table 1), indicated that this species was present quite frequently in spring (Figure 7C), even though we were not able to detect it by QPS.

## Discussion

CARD-FISH was tested extensively on ciliates from cultures and on ciliates from water samples of Lake Zurich. Using a custom-modified protocol, we were able to identify and quantify planktonic ciliates in field samples by fluorescence *in situ* hybridization for the first time.

### Probe specificity and accuracy of quantification by CARD-FISH

After some adjustments, all oligonucleotide probes tested on ciliate cultures were specific. Diluting ciliate cultures in the mock assemblage with lake water did not affect the number of hybridized cells, further illustrating the specificity of the examined oligonucleotide probes. The only probe in this study with an *in silico* ‘outgroup’ hit was Hal-all-1362, designed to be specific for the genus *Halteria*: A closely related freshwater ciliate species, *Meseres corlissi*, is also targeted by the probe (Supplementary Figure S2). However, this species is rare and prefers temporarily flooded habitats, such as small meadow ponds, floodplain soils, and tanks of bromeliads (Weisse et al., 2008), and it has never been found in previous studies of Lake Zurich (Posch et al., 2015; Pitsch et al., 2019; Forster et al., 2021). Moreover, observations from QPS filters indicated that “*Halteria*” cells with algal endosymbionts probably belonged to the species *Pelagohalteria viridis*. Since the probe Hal-all-1362 also hybridized with cells harboring endosymbionts, it is likely that it also covers the genus *Pelagohalteria*. To date, no 18S rRNA gene sequence of this morphologically very similar genus is available in public databases, and it is therefore not possible to discriminate it from the genus *Halteria* by CARD-FISH. The unclear specificity together with the poor preservation of cells (see below) may explain the discrepancy between counts by QPS and CARD-FISH at some time points (Figure 7C).

The discrimination between the three species *U. cf. nais*, *U. cf. pseudofurcata,* and *U. cf. castalia* provides an example for the high specificity of the newly designed probes and it allowed us to reliably quantify *U. cf. pseudofurcata* in water samples from Lake Zurich. So far, the limited morphological differences have made it difficult to distinguish between small *Urotricha* species by QPS. By contrast, even the tiny *U. cf. pseudofurcata* cells could be readily detected on CARD-FISH filters due to their intense fluorescent signal. The same holds true for cells hybridized with the genus-specific probe Uro-all-651. The strikingly good agreement between QPS and CARD-FISH cell counts of small (≤ 35 μm) *Urotricha* spp. indicated that the genus-specific probe likely covered most members of this genus in Lake Zurich. One reason for this encouraging finding are the recently published 18S rRNA gene sequences of five *Urotricha* species (Frantal et al., 2022; Sonntag et al., 2022), that were included in the design of probe Uro-all-651. At the same time, the poor agreement between QPS and CARD-FISH in the sample from April 8, 2020 indicates that not all *Urotricha* species found in Lake Zurich may be covered by the probe. Besides *Urotricha* spp.*, C. margaritaceum* was also accurately quantified in lake samples by CARD-FISH. Although both methods allowed for a precise determination of the population sizes of this species, its quantification was much easier and faster with CARD-FISH than with QPS.

### CARD-FISH hybridization signal

The CARD-FISH protocol used in this study resulted in a distinct, bright fluorescent signal of hybridized cells. Together with the low background fluorescence, this allowed for a straightforward recognition and counting of the stained ciliates, including the rare ones, in water samples harboring thousands of other eukaryotic organisms.

An obstacle in the study of CARD-FISH filters from lake water arose from the presence of centric diatoms (Figure 6S). They emitted a strong fluorescence signal, similar to hybridized ciliates owing to unspecific binding of oligonucleotide probes and/or tyramides. The same phenomenon has also been observed in other studies (e.g., Piwosz et al., 2021). Owing to their characteristic appearance (shape, frustule and nucleus), diatoms were nevertheless easily distinguishable from hybridized ciliates and only made ciliate counts challenging if present in very high numbers.

### Problems encountered during quantification

A major limitation for the quantification of ciliates using CARD-FISH was the poor preservation of some species, i.e., *H. grandinella* and *M. chlorelligerum*. In particular, many cells of *H. grandinella* cells already disintegrated during filtration. Tests with Lugol’s solution in combination with formaldehyde, a frequently used fixative for CARD-FISH with protists (e.g., Piwosz et al., 2021), did not improve these result. Osmium tetroxide (OsO_4_), a fixative previously used for CARD-FISH of ciliates (Petroni et al., 2003), was not tested in this study. Due to its high toxicity, it was considered unsuitable for the routine use in large volumes of field samples. Another powerful fixative for ciliates, Bouin’s solution (50% f.c.), has successfully been applied for FISH before, resulting in good cell preservation and high detection rate of hybridized cells (Fried et al., 2002; Fried and Foissner, 2007; Zhan et al., 2018). However, our own preliminary tests with several ciliate species indicated that this fixing agent was incompatible with our CARD-FISH protocol; it induced an intense green fluorescence signal even in the absence of oligonucleotide probes, thereby impairing the differentiation of hybridized ciliates. We thus advise against the use of Bouin’s solution as fixative for CARD-FISH of ciliates. In contrast to previous studies (Stoecker et al., 1994; Fried et al., 2002; Karayanni et al., 2004; Modigh and Castaldo, 2005), the best fixation was achieved using formaldehyde (1.75–3.5% f.c.), allowing for accurate quantification of six of the eight examined ciliates. Other fixatives such as ethanol (Schmidt et al., 2006) or buffered paraformaldehyde solution (Fried et al., 2002) should be examined to further improve the preservation of delicate ciliate species for CARD-FISH.

Our results from *M. chlorelligerum* indicated that the CARD-FISH procedure itself could lead to cell loss. Although the low density of these cultures hampered accurate quantification, it was nevertheless striking how few cells were found on the filter after the procedure. It is conceivable that the spherical cells of *M. chlorelligerum* were lost from the filters during the washing and hybridization steps in the tubes. The use of higher agarose concentration did not improve these results. A possible remedy might be to embed filters in a thicker layer of agarose or to apply a gentler CARD-FISH method (Pernthaler and Pernthaler, 2007).

### Choice of filter set for detection by epifluorescence microscopy

FISH preparations of cultured algivorous ciliates always included numerous cells of their primary food source, *Cryptomonas* sp.. During initial microscopic evaluation of FISH filters with Zeiss filter set 10 (Supplementary Table S4), we found that these *Cryptomonas* cells also emitted strong green fluorescence, even if partially digested inside the food vacuoles of ciliates (Bühler, 2020). Thus, it was very difficult to distinguish between hybridized, fluorescently labeled ciliate cells and the autofluorescence of *Cryptomonas* sp. or other algae found in Lake Zurich (e.g., *Pediastrum* sp., *Uroglena* sp.). This issue could eventually be overcome by changing to Zeiss filter set 62 HE (Supplementary Table S4). Using this filter set, autofluorescence of algae appeared clearly red when excited with blue light and could therefore be easily distinguished from the green emission of hybridized cells.

### *Balanion planctonicum*—a challenging species

*Balanion planctonicum* is a frequent, temporarily highly abundant ciliate species found worldwide in temperate lakes and ponds (Müller, 1991; Macek et al., 1996; Modenutti and Pérez, 2001; Munawar and Lynn, 2002; Sonntag et al., 2006; Li et al., 2013; Kammerlander et al., 2016). In Lake Zurich, the tiny ciliate is usually common during spring and early summer (Posch et al., 2015; Pitsch et al., 2019). Counts from QPS filters revealed that this was also the case for the year 2020. By applying CARD-FISH with the same water samples, we rarely found hybridized *B. planctonicum* cells, whereas hybridizations on *B. planctonicum* cultures worked perfectly. Therefore, the common reasons for the false negative results in field samples are unlikely, such as poor cell preservation, imperfect probe coverage and specificity, lack of cell permeabilization or low efficiency of probe binding due to higher-order structure of the rRNA (Pernthaler et al., 2004; Amann and Fuchs, 2008; Piwosz et al., 2021). Another reason for the observed discrepancy could be a weak, non-contrasting fluorescence signal of hybridized cells due to lower concentration of rRNA during the stationary growth phase (Lim et al., 1993; Fu and Gong, 2017). However, this unlikely explains the low CARD-FISH detection rates of *B. planctonicum* in lake samples. For one, their high numbers, as found by QPS, indicated rather high growth rates. Secondly, previous studies have reported that CARD-FISH should allow for the detection of picoeukaryotic cells both, during exponential growth and stationary phase (Biegala et al., 2003). Finally, there were few cells in the lake water samples that indeed emitted a bright fluorescent signal after CARD-FISH (Figure 6K). Thus a more likely explanation might be that our probes only matched to the ribosomal RNA of a small subpopulation, whereas the majority of cells, considered to be *B. planctonicum* based on morphological analysis, were genetically distinct from our cultivated strains. Results from a previous study already hinted at variability within the V9 region of the 18S rRNA gene sequence of *B. planctonicum* from Lake Zurich (Pitsch et al., 2019). Consequently, ciliates classified as *B. planctonicum* by QPS might represent a morphospecies harboring cryptic taxa, or might even consist of several species. Only small differences (two mismatches) were detected in the 18S rDNA sequences of three separately isolated strains. In order to elucidate the *in situ* genotypic variability of *B. planctonicum* in Lake Zurich, we are currently collecting complete 18S rRNA sequences affiliated with this species without prior cultivation.

### Application of CARD-FISH to study freshwater ciliates

Our results confirm and expand previous studies showing a possible distinction between closely related (Petroni et al., 2003; Schmidt et al., 2006; Fried and Foissner, 2007) or even cryptic species (Melekhin et al., 2022) by specific oligonucleotide probes. In addition, the rapid processing of numerous samples by our approach allows for both, studies at high temporal resolution and for long-term monitoring programs. Large sample volumes in combination with the bright fluorescent signal of hybridized cells facilitates the detection of rare and/or barely active cells (Hoshino et al., 2008). Furthermore, the possibility of double hybridization offers the opportunity to study the ecological role of specific ciliate species or genera in lacustrine pelagic environments. By combining two or more probes, protists at different trophic levels can be simultaneously targeted, i.e., the predator and its prey inside food vacuoles (Massana et al., 2009; Piwosz and Pernthaler, 2010; Grujcic et al., 2018; Šimek et al., 2020). Thus, CARD-FISH could be a powerful tool for studying the *in situ* food preferences of, e.g., the omnivorous genus *Urotricha*. Representatives of this genus are widespread and abundant inhabitants of freshwater environments (Müller, 1989; Posch et al., 2015; Šimek et al., 2019; Frantal et al., 2022; Sonntag et al., 2022), but little is known about their feeding strategies *in situ* (Müller et al., 1991b; Sonntag et al., 2006; Qu et al., 2021). In addition to food selectivity, CARD-FISH also provides the potential to further examine the symbiotic relationships within eukaryotic and between eukaryotic and prokaryotic microbes (Chambouvet et al., 2008; Dirren and Posch, 2016; Lepère et al., 2016). Although ciliates have been known to harbor endo- and ectosymbionts since the late 19th century, little is known about the ecological function or the *in situ* dynamics of such relationships in the field (Dziallas et al., 2012). In summary, our quantitative CARD-FISH approach might provide a means to profit from the rapidly increasing number of available probes for different protistan lineages (summarized in Piwosz et al., 2021), and to study the dynamics and interactions of freshwater pelagic ciliates.

## Data availability statement

The datasets presented in this study can be found in online repositories. The names of the repository/repositories and accession number(s) can be found in the article/Supplementary material.

## Author contributions

Ciliates were isolated and cultivated by GD-P and BB. DB, JP, MMS, and TP contributed to the design of the study. MMS designed the oligonucleotide probes for *in situ* hybridization. Lab work was primarily done by DB and BB. AM did the statistical analysis. QPS data was evaluated by MS. GD-P produced all figures and the first version of the manuscript, with input from all authors. All authors contributed to the article and approved the submitted version.

## Funding

This study was supported by the Swiss National Science Foundation grants 31003A_182489 and 31003A_182336. MMS was funded by the Czech Science Foundation (GACR) grant 20-12496X. ALM was supported by the University Research Priority Program “Global Change and Biodiversity” of the University of Zürich.

## Conflict of interest

The authors declare that the research was conducted in the absence of any commercial or financial relationships that could be construed as a potential conflict of interest.

## Publisher’s note

All claims expressed in this article are solely those of the authors and do not necessarily represent those of their affiliated organizations, or those of the publisher, the editors and the reviewers. Any product that may be evaluated in this article, or claim that may be made by its manufacturer, is not guaranteed or endorsed by the publisher.
