# Supplementary material for: FISHing for ciliates: Catalyzed reporter deposition fluorescence in situ hybridization for the detection of planktonic freshwater ciliates
Source: Front Microbiol. 2022 Dec 12;13:1070232. doi: 10.3389/fmicb.2022.1070232 (PMC9790926; doi:10.3389/fmicb.2022.1070232)
Supplement: Supplementary file 1 [file Table_1.DOCX]

**Table S1:** Forward and reverse primers used to amplify the 18S rDNA sequence of six studied ciliate species. Note that for some species two forward primers were needed for the amplification of the 18S rDNA.

| **Forward primer** | **Sequence (5' to 3')** | **Reference** | **Used for ciliate species** |
| --- | --- | --- | --- |
| EAF3 | TCGACAATCTGGTTGATCCTGCCAG | Marin et al., 2003 | *Askenasia* cf. *volvox* |
| EUKA | ACCTGGTTGATCCTGCCAG | Medlin et al., 1988 | *Monodinium chlorelligerum* |
|  |  |  | *Balanion planctonicum* |
|  |  |  | *Urotricha* cf. *pseudofurcata* |
|  |  |  | *Halteria* cf. *bifurcata* |
|  |  |  | *Halteria grandinella* |
| SR4 | AGCCGCGGTAATTCCAGCT | Nakayama et al. 1998 | *Monodinium chlorelligerum* |
|  |  |  | *Balanion planctonicum* |
|  |  |  | *Urotricha* cf. *pseudofurcata* |
| SR8 | GGATTGACAGATTGAGAGCT | Nakayama et al. 1998 | *Halteria* cf. *bifurcata* |
|  |  |  |  |
| **Reverse primer** | **Sequence (5' to 3')** | **Reference** |  |
| n1400R | GGTAGGAGCGACGGGCGGTGTGTAC | Marin et al., 2003 | *Askenasia* cf. *volvox* |
| EUKB | TGATCCTTCTGCAGGTTCAC | Medlin et al., 1988 | *Monodinium chlorelligerum,* |
|  |  |  | *Balanion planctonicum,* |
|  |  |  | *Urotricha* cf. *pseudofurcata,* |
|  |  |  | *Halteria* cf. *bifurcata* |
|  |  |  | *Halteria grandinella* |

**References**

Marin, B., Palm, A., Klingberg, M.A.X., and Melkonian, M. (2003). Phylogeny and taxonomic revision of plastidcContaining euglenophytes based on SSU rDNA sequence comparisons and synapomorphic signatures in the SSU rRNA secondary structure. Protist 154, 99-145. doi: 10.1078/143446103764928521

Medlin, L., Elwood, H.J., Stickel, S., and Sogin, M.L. (1988). The characterization of enzymatically amplified eukaryotic 16S-like rRNA-coding regions. Gene 71, 491-499. doi: 10.1016/0378-1119(88)90066-2

Nakayama, T., Marin, B., Kranz, H.D., Surek, B., Huss, V.A.R., Inouye, I., et al. (1998). The basal position of scaly green flagellates among the green algae (Chlorophyta) is revealed by analyses of nuclear-encoded SSU rRNA sequences. Protist 149, 367-380. doi: 10.1016/S1434-4610(98)70043-4
