# Supplementary material for: FISHing for ciliates: Catalyzed reporter deposition fluorescence in situ hybridization for the detection of planktonic freshwater ciliates
Source: Front Microbiol. 2022 Dec 12;13:1070232. doi: 10.3389/fmicb.2022.1070232 (PMC9790926; doi:10.3389/fmicb.2022.1070232)
Supplement: Supplementary file 2 [file Table_2.DOCX]

**Table S2:** Oligonucleotide probes used for CARD-FISH. The name and sequence of species-, genus-, as well as the eukaryote-specific probes, their optimal formamide concentrations in hybridization buffer (FA in %), their competitors and corresponding sequences are listed. Reference of all probes: this study.

| **Species** | **Probe** | **FA [%]** | **Competitors** | **Sequence (5' - 3)** |
| --- | --- | --- | --- | --- |
| *Askenasia* cf. *volvox* | Ask-193 | 60 |  | TTAGCGAGGCGAACCTCGCTTCGATCAGT |
|  |  |  | ask-193-C1 | TTAGCGAGGTAAACCTCGCTTCGATCAGT |
|  |  |  | ask-193-C2 | TTAGCGAGGCWAGCCTCGCTTCGATCAGT |
| *Monodinium chlorelligerum* | MonoZH-179 | 60 |  | TACGCCCTTGCGGGCTTGG |
| *Monodinium* spp. (genus) | Mono-all-826 | 60 |  | CGAATCCAWACCAATCCCTAG |
| *Cinetochilum margaritaceum* | Cin-1237 | 60-70 |  | GAAATACTCGCTGCACGTGTCATTG |
|  |  |  | cin-1237-C1 | GAAATACTCGCTGAATGTGTCATTG |
|  |  |  | cin-1237-C2 | GATATACTCGCTGCACGTGTCATTG |
|  |  |  | cin-1237-C3 | GAAAAACTCGCTGCACGTGTCATTG |
| *Balanion planctonicum* | Bal-651 | 60-70 |  | TGAAGACCGAACGGAGTTCCC |
|  |  |  | bal-651-C1 | TGAAGTTCGAACGGAGTTCCC |
|  |  |  | bal-651-C2 | TGAAGTCCGAACGTAGTTCCC |
|  |  |  | bal-651-C3 | TGAAGACCGAACGGGGTTCCC |
|  |  |  | bal-651-C4 | TGAAGACCGAACAGGGTTCCC |
|  |  |  | bal-651-C5 | TGAAGACCGAACGAGGTTCCC |
|  |  |  | bal-651-C6 | TGAAGGACGAACGGAGTTCCC |
| *Urotricha* cf. *castalia* | Uro2-1440 | 60-70 |  | CGTTGACTCAAGGACAACGACGGTCCAG |
| *Urotricha* cf. *nais* | Uro4-1436 | 60 |  | AACTCAAGGAACTGTACAGTCCAGAAGG |
|  |  |  | uro4-1436-C | AACTCAAGGATCTGTACGGTCCAGAAGG |
| *Urotricha* cf. *pseudofurcata* | Uro5-403 | 60 |  | TGAAAGGACCCCGAGTTGTT |
| *Urotricha* spp. (genus) | Uro-all-651 | 60 |  | ATGACGATTGCCTGCCTTGAA |
|  |  |  | uro-all-651-C1 | ATGGCGATTGCCTGCCTTGAA |
|  |  |  | uro-all-651-C2 | ATGACGATTGCCTGCCGTGAA |
|  |  |  | Bal-651-C ^a)^ | TGAAGACCGAACGGAGTTCCC |
| *Halteria* cf. *bifurcata* | HalZH-1133 | 65 |  | AGTCGGGGCCAATCTAGGATTGG |
|  |  |  | halZH-1133-C1 | AGTCGCGGCCAATCTAGGATTGG |
| *Halteria* spp. (genus) | Hal-all-1362 | 50 |  | CAGGGACTTAATCGGCGCAAGGTA |
|  |  |  | hal-all-1362-C1 | CAGGGACTTAATCGACGCAAGGTA |
|  |  |  | hal-all-1362-C2 | CAGGGACTTAATCAGCGCAAGGTA |
|  |  |  | hal-all-1362-C3 | CAGGGACTTAATCGGCGCAAGCTA |
| Eukaryote-specific probe | EUK1209-mod | 60 |  | GGGCATCACWGACCTGTT |

1. Probe Uro-all-651 initially gave a false positive signal by also hybridizing *B. planctonicum* cells. This could be prevented by including the oligonucleotide Bal-651 as an additional competitor.
