# Supplementary material for: FISHing for ciliates: Catalyzed reporter deposition fluorescence in situ hybridization for the detection of planktonic freshwater ciliates
Source: Front Microbiol. 2022 Dec 12;13:1070232. doi: 10.3389/fmicb.2022.1070232 (PMC9790926; doi:10.3389/fmicb.2022.1070232)
Supplement: Supplementary file 3 [file Table_3.DOCX]

**Table S3:** Filters, solutions and buffers used for CARD-FISH. For further information about recipes for buffers, solutions and reagents see Supplementary Files in Piwosz et al. (2021).

**Filters, solutions and buffers for CARD-FISH**

- **Formaldehyde** solution (37%, Carl Roth, Germany)
- **Polycarbonate membrane filters:**
  - TSTP with a pore size of **3 µm** (diameter: 25 mm & 47mm, Isopore™, Merck Millipore, Germany)
  - RTTP with a pore size of **1.2 µm** (diameter: 25 mm & 47mm, Isopore™, Merck Millipore, Germany)
- **Support filters:**
  - Cellulose nitrate membrane filter AE 99 with a pore size of 8 µm (diameter: 50 mm, Schleicher & Schuell, Germany)
  - Cellulose nitrate membrane filter with a pore size of 5 µm (diameter: 25 mm, Sartorius, Germany)
- **1x PBS** (1x phosphate buffered saline, pH 7.6)
- **Agarose** (Standard Agarose, Type LE, BioConcept, Switzerland)
- **Oligonucleotide probes for CARD-FISH** 5’ tagged with a HRP (horseradish peroxidase) (biomers.net GmbH, Germany)
- **Hybridization buffer** 20 ml:
  - 3600 µl 5M NaCl
  - 400 µl 1M Tris-HCl (pH 7.4)
  - 2 g dextran sulfate 500 (Carl Roth, Germany)
  - Different volumes of formamide (Rotipuran ≥ 99.5%, Carl Roth, Germany) and deionized sterile water depending on the intended final formamide concentration (Suppl. Table S2, Piwosz et al. 2021)
  - 2000 µl 10% blocking reagent (50 ml of 10% blocking reagent consist of: 0.438 g 0.15M NaCl, 0.5805 g 0.1M maleic acid, 50 ml deionized sterile water, 5 g blocking reagent (Roche Diagnostics, Switzerland))
  - 20 µl 10% SDS (sodium dodecyl sulfate)
- **Washing buffer** 50 ml:
  - 500 µl 0.5M EDTA (pH 8)
  - 1000 µl 1M Tris-HCl (pH 7.4)
  - Different volumes of 5M NaCl, depending on formamide concentration of the hybridization buffer (Suppl. Table S2, Piwosz et al. 2021)
  - filled up with deionized sterile water to a volume of 50 ml
  - 50 µl 10% SDS (sodium dodecyl sulfate)
- **PBST** = PBS/Triton X-100 solution (1x phosphate-buffered saline, 0.1% Triton X-100 (Merck Sigma-Aldrich, Germany), pH 7.6)
- **Tyramide solution mixture** (consist of 3 ml amplification buffer, 30 µl 0.15% H_2_O_2_ and 3 µl fluorochrome-labeled tyramides)
  - **Amplification buffer**
    - 4 ml 10x PBS (10x phosphate-buffered saline)
    - 16 ml 5M NaCl
    - 4 g dextran sulfate 500 (Carl Roth GmbH, Germany)
    - 0.4 ml 10% Blocking reagent (Roche Diagnostics GmbH, Germany)
    - filled up with deionized sterile water to a volume of 40 ml
  - **0.15% H_2_O_2_**
    - 5 µl 30% H2O2 (Merck Sigma-Aldrich, Germany)
    - 1 ml 1x PBS
  - **Fluorochrome-labeled tyramides**
    - 3.3 ml Tyramide stock
    - 3 ml dimethylformamide (Merck Sigma-Aldrich, Germany)
    - 33 µl triethylamine (Merck Sigma-Aldrich, Germany)
    - 33 mg Tyramine-HCl (Merck Sigma-Aldrich, Germany)
    - 10 ml NHS-Carbofluorescein (to be prepared on ice and in the dark)
    - 100 mg NHS-Fluorescein (or Alexa Fluor™ 546 NHS Ester) (ThermoFisher, USA)
    - 10 ml dimethylformamide (Merck Sigma-Aldrich, Germany)
    - Tyramide stock and NHS-Carbofluorescein are mixed and incubated at room temperature in the dark for 12 h, storage at -20 °C or desiccated for long term storage (resuspension with dimethylformamide containing 20 mg ml^-1^ p-iodophenylboric acid)
- **DAPI-Mix** (4’, 6’-diamidino-2-phenylindole – mix, pH 9):
  - 5x Glycerol Citiflour AF1 (Linaris Biologische Produkte, Germany)
  - 1x Vectashield (Linaris Biologische Produkte, Germany)
  - 1x PBS
  - 1 µg DAPI ml^-1^ (Merck Sigma-Aldrich, Germany)

**References**

Piwosz, K., Mukherjee, I., Salcher, M.M., Grujčić, V., and Šimek, K. (2021). CARD-FISH in the sequencing era: Opening a new universe of protistan ecology. *Front. Microbiol.* 12:640066. doi: 10.3389/fmicb.2021.640066
