# Supplementary material for: FISHing for ciliates: Catalyzed reporter deposition fluorescence in situ hybridization for the detection of planktonic freshwater ciliates
Source: Front Microbiol. 2022 Dec 12;13:1070232. doi: 10.3389/fmicb.2022.1070232 (PMC9790926; doi:10.3389/fmicb.2022.1070232)
Supplement: Supplementary file 4 [file Table_4.DOCX]

**Table S4:** Different filter sets used for epifluorescence microscopy using the Zeiss Axio Imager.M1 microscope. HE = high efficiency filter set, BP = band pass filter, FT = color splitter, LP = long pass filter.

| **Filter set (ZEISS)** | **Excitation** | **Beamsplitter** | **Emission** | **Visualization of e.g.** |
| --- | --- | --- | --- | --- |
| Filter Set 43 | BP 545/25 | FT 570 | BP 605/70 | Fluorochrome Alexa Fluor 546 |
| Filter Set 62 HE (Colibri) | BP 370/40 (HE) | TFT 395 + 495 + 610 (HE) | TBP 425 + 527 + LP615 (HE) | Fluorochrome Fluorescein Isothiocyanate (FITC) |
|  | BP 474/28 (HE) |  |  |  |
|  | BP 585/35 (HE) |  |  |  |
| Filter Set 10 | BP 450-490 | FT 510 | BP 515-565 | Fluorochrome Fluorescein Isothiocyanate (FITC) |
| Filter Set 01 | BP 365/12 | FT 395 | LP 397 | DAPI-stained DNA |
