# Supplementary material for: FISHing for ciliates: Catalyzed reporter deposition fluorescence in situ hybridization for the detection of planktonic freshwater ciliates
Source: Front Microbiol. 2022 Dec 12;13:1070232. doi: 10.3389/fmicb.2022.1070232 (PMC9790926; doi:10.3389/fmicb.2022.1070232)
Supplement: Supplementary file 5 [file Table_5.docx]

**Table S5:** Details of the statistical analysis of the comparison of cell counts between live, DAPI and CARD-FISH for eight different ciliate cultures (Fig. 4 and Suppl. Fig. S4). Parametric and non-parametric tests were chosen according to the normality and homoscedasticity of the dataset. P-values were adjusted for false discovery rate due to multiple testing with the Benjamini-Hochberg (1995) correction. Results with an adjusted p-value close to the α=0.05 threshold are highlighted in bold and were considered for post-hoc analyses (Suppl. Table S6).

| **Kruskal-Wallis** |  |  |  |  |  |
| --- | --- | --- | --- | --- | --- |
| **Species** | **H** | **df** | **p-value** | **adj. p-value** |  |
| *A. cf*. *volvox* | 1.76 | 2 | 0.41 | 0.47 |  |
| *H. grandinella* | 8.07 | 2 | 0.02 | **0.07** |  |
|  |  |  |  |  |  |
| **ANOVA** |  |  |  |  |  |
| **Species** | **F** | **df1** | **df2** | **p-value** | **adj. p-value** |
| *M. chlorelligerum* | 7.08 | 2 | 5 | 0.03 | **0.07** |
| *C. margaritaceum* | 5.95 | 2 | 7 | 0.03 | **0.07** |
| *B. planctonicum* | 1.31 | 2 | 10 | 0.31 | 0.42 |
| *U*. *cf*. ps*eudofurcata* | 2.22 | 2 | 12 | 0.15 | 0.24 |
| *U. cf*. *castalia* | 6.54 | 2 | 15 | 0.01 | **0.07** |
| *U. cf*. *nais* | 0.72 | 2 | 8 | 0.52 | 0.52 |

**Reference**

Benjamini, Y., and Hochberg, Y. (1995). Controlling the false discovery rate: A practical and powerful approach to multiple testing. *J. R. Stat. Soc., B: Stat. Methodol*. 57**,** 289-300. doi: 10.1111/j.2517-6161.1995.tb02031.x
