# Supplementary material for: FISHing for ciliates: Catalyzed reporter deposition fluorescence in situ hybridization for the detection of planktonic freshwater ciliates
Source: Front Microbiol. 2022 Dec 12;13:1070232. doi: 10.3389/fmicb.2022.1070232 (PMC9790926; doi:10.3389/fmicb.2022.1070232)
Supplement: Supplementary file 6 [file Table_6.docx]

**Table S6:** Details of the post-hoc analyses of the comparisons of cell counts between live, DAPI and CARD-FISH for the four ciliate cultures displaying an adjusted p-value close to the α=0.05 threshold (Fig. 4, Suppl. Fig. S4, Suppl. Table S5). Significant results are highlighted in bold.

| **Species** | **Post Hoc Test** | **adj. p-value** | | |
| --- | --- | --- | --- | --- |
|  |  | **DAPI-CARD** | **live-CARD** | **live-DAPI** |
| *M. chlorelligerum* | Tukey’s HSD | 0.20 | **0.03** | 0.40 |
| *C. margaritaceum* | Tukey’s HSD | **0.03** | 0.07 | 0.77 |
| *U. cf. castalia* | Tukey’s HSD | 0.86 | 0.11 | **0.01** |
| *H. grandinella* | Dunn's Test | 0.11 | **0.01** | 0.10 |
