# Supplementary material for: FISHing for ciliates: Catalyzed reporter deposition fluorescence in situ hybridization for the detection of planktonic freshwater ciliates
Source: Front Microbiol. 2022 Dec 12;13:1070232. doi: 10.3389/fmicb.2022.1070232 (PMC9790926; doi:10.3389/fmicb.2022.1070232)
Supplement: Supplementary file 7 [file Table_7.docx]

**Table S7:** Details of the statistical analysis of the comparison of cell counts between live, DAPI and CARD-FISH for *H. grandinella* and *M. chlorelligerum* under different fixation and CARD-FISH methods at various dates (see Suppl. Fig. S5). Parametric and non-parametric tests were chosen according to the normality and homoscedasticity of the dataset. P-values were adjusted for false discovery rate due to multiple testing with the Benjamini-Hochberg (1995) correction. Results with an adjusted p-value <0.05 or close to the α=0.05 threshold are highlighted in bold and were considered for post-hoc analyses (Suppl. Table S8).

| **Kruskal-Wallis** |  |  |  |  |  |  |
| --- | --- | --- | --- | --- | --- | --- |
| **Species** | **Treatment** | **H** | **df** | **p-value** | **adj. p-value** |  |
| *H. grandinella (17.1)* | Formaldehyde | 8.07 | 2 | 0.02 | **0.07** |  |
|  |  |  |  |  |  |  |
| **ANOVA** |  |  |  |  |  |  |
| **Species** | **Treatment** | **F** | **df1** | **df2** | **p-value** | **adj. p-value** |
| *H. grandinella (17.1)* | Lugol- Formaldehyde | 127.38 | 2 | 7 | 10^-6^ | **10^-5^** |
| *H. grandinella (12.1)* | Formaldehyde | 22.89 | 2 | 6 | 0.002 | **0.002** |
| *H. grandinella (12.1)* | Lugol- Formaldehyde | 153.82 | 2 | 6 | 10^-5^ | **10^-5^** |
| *M. chlorelligerum (11.1)* | Formaldehyde | 39.81 | 2 | 5 | 10^-4^ | **0.002** |
| *M. chlorelligerum (19.1)* | Formaldehyde | 7.08 | 2 | 5 | 0.03 | **0.07** |
| *M. chlorelligerum (19.1)* | Lugol- Formaldehyde | 6.45 | 2 | 5 | 0.04 | **0.04** |
| *M. chlorelligerum (19.1)* | Formaldehyde, 0.3% Agar | 8.97 | 2 | 5 | 0.02 | **0.03** |

**Reference**

Benjamini, Y., and Hochberg, Y. (1995). Controlling the false discovery rate: A practical and powerful approach to multiple testing. *J. R. Stat. Soc., B: Stat. Methodol*. 57**,** 289-300. doi: 10.1111/j.2517-6161.1995.tb02031.x
