# Supplementary material for: FISHing for ciliates: Catalyzed reporter deposition fluorescence in situ hybridization for the detection of planktonic freshwater ciliates
Source: Front Microbiol. 2022 Dec 12;13:1070232. doi: 10.3389/fmicb.2022.1070232 (PMC9790926; doi:10.3389/fmicb.2022.1070232)
Supplement: Supplementary file 8 [file Table_8.docx]

**Table S8:** Details of the post-hoc analyses of the comparisons of cell counts between live, DAPI and CARD-FISH for H. grandinella and M. chlorelligerum under different fixation and CARD-FISH methods at various dates (in brackets, see Fig. S5, Suppl. Table S7). Significant results are highlighted in bold.

| **Species** | **Treatment** | **Post Hoc Test** | **adj. p-value** | | |
| --- | --- | --- | --- | --- | --- |
|  |  |  | **DAPI-CARD** | **live-CARD** | **live-DAPI** |
| *H. grandinella (17.1)* | Formaldehyde | Dunn's Test | 0.11 | **0.007** | 0.10 |
| *H. grandinella (17.1)* | Lugol- Formaldehyde | Tukey's HSD | **10^-4^** | **10^-6^** | **10^-4^** |
| *H. grandinella (12.1)* | Formaldehyde | Tukey's HSD | **0.004** | **0.001** | 0.53 |
| *H. grandinella (12.1)* | Lugol- Formaldehyde | Tukey's HSD | **0.01** | **10^-6^** | **10^-5^** |
| *M. chlorelligerum (11.1)* | Formaldehyde | Tukey's HSD | **0.005** | **10^-4^** | 0.18 |
| *M. chlorelligerum (19.1)* | Formaldehyde | Tukey's HSD | 0.20 | **0.03** | 0.40 |
| *M. chlorelligerum (19.1)* | Lugol- Formaldehyde | Tukey's HSD | 0.29 | **0.04** | 0.33 |
| *M. chlorelligerum (19.1)* | Formaldehyde, 0.3% Agar | Tukey's HSD | 0.12 | **0.02** | 0.40 |
