# Supplementary material for: FISHing for ciliates: Catalyzed reporter deposition fluorescence in situ hybridization for the detection of planktonic freshwater ciliates
Source: Front Microbiol. 2022 Dec 12;13:1070232. doi: 10.3389/fmicb.2022.1070232 (PMC9790926; doi:10.3389/fmicb.2022.1070232)
Supplement: Supplementary file 9 [file Data_Sheet_1.PDF]

CARD-FISH for Ciliates (1/3)

	Instructions	Additional Information
**Fixation:** 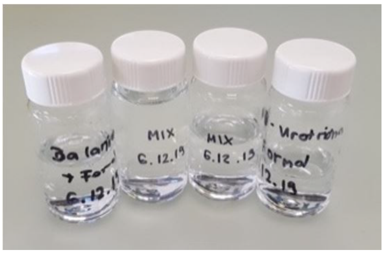	Fix x ml sample with formaldehyde (1.76% f.c.). Incubate fixed samples for 1 h at 4 °C in the dark.	Use glass tubes for fixation. Plastic tubes will reduce the number of fixed cells as they may stick to the wall.
**Filtration:** 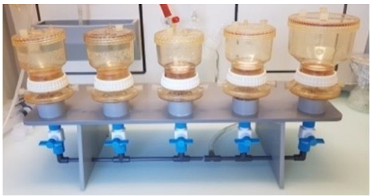	Prepare filter towers: rinse thoroughly with deionized sterile water and equip them with support and polycarbonate membrane (PC) filters.<br>Filter x ml fixed sample gently onto PC filters with low pressure (<50 mbar) and wash twice with ~10 ml PBS.<br>Label filters with a pencil and air dry them before storing at -20 °C.	Using too high pressure can destroy cells.<br>In order to ensure an even distribution of organisms, i) filter slowly and ii) let the PBS run along sides of the filtration tower while washing.<br>Alternatively, filters can be stored at -20 °C until further processing after the next step (embedding in agarose).
**Embedding in agarose:** 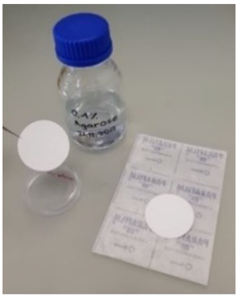	Melt 0.1% agarose in microwave oven and cool it down to approx. 40 °C.<br>Dip filters into agarose and dry them face up on a parafilm at 37 °C for about 30 min.	Make sure that there is only a thin and even layer of agarose on the filter.<br>▶ Embedding in agarose minimizes cell loss.
**Incubation in HCl:** 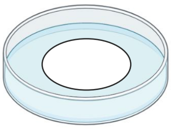 HCl	Incubate filters in 0.01M HCl for 20 min.	Incubation in HCl reduces background fluorescence and inhibits endogenous peroxidases.

Figure S3: Detailed CARD-FISH protocol, elaborated from cultivated ciliates as well as lake water samples. A detailed list of used chemicals and materials is shown in Table S3. Figures of Petri dishes, Falcon tube, glass slide and microscope were created with BioRender.com (2021).

Washing:

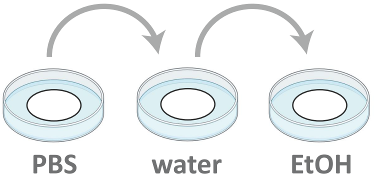

Wash filters with PBS, deionized sterile water and EtOH for about 1 min each. Put them on a blotting paper until they are dry.
Filters can be stored at -20 °C.

If less chlorophyll fluorescence is desired, prolong the incubation time in EtOH to 10 min.

Cut filters into pieces:

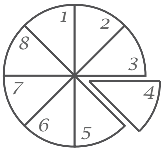

Label filters with a pencil and cut them into pieces.

Be careful not to damage filters.

Hybridization:

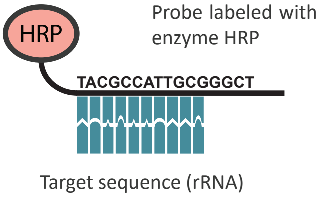

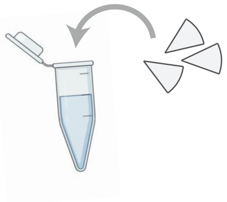

For filters with 25 mm Ø:
Mix 300 µL hybridization buffer, 2 µl probe (50 µg ml^{-1}) and 2µl competitor if required (50 µg ml^{-1}) in a 0.5 ml Eppendorf tube.

For filters with 47 mm Ø:
Mix 900 µl hybridization buffer, 6 µl probe (50 µg ml^{-1}) and 6µL competitor if required (50 µg ml^{-1}) in a 1.5 ml Eppendorf tube.

Put filter sections into this mixture and place them into a rotary oven at 35 °C for 3 h.

Make sure that all filters are moistened with the hybridization solution during this procedure.
▶ HRP-labeled oligonucleotide probes bind to the targeted ribosomal RNA.
▶ The higher the formamide concentration in the hybridization buffer, the more specific the binding of the probe to the target sequence.

Washing:

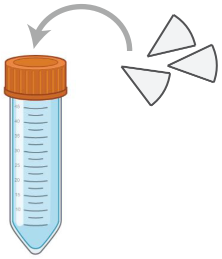

To obtain washing buffer, add the needed solutions (Table S3) in a 50 ml falcon tube. Mix by tilting it regularly in a water bath at 37 °C.
Put filters into the preheated washing buffer and wash them for 30 min at 37 °C in the dark.

Depending on the utilized formamide concentration, different amounts of NaCl have to be added in the washing buffer (Table S3).

Incubation in PBST:

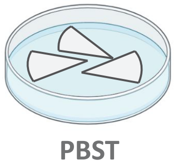

Place filters for 45 min in PBST at 37 °C in the dark. Use a shaker (about 75 RMP).

PBST equilibrates the probe-delivered HRP.

Figure S3: Continued

Tyramide signal amplification:

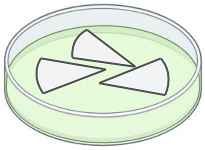

Prepare tyramide solution mixture in a small petri dish. Remove filters from PBST and put them onto blotting paper to remove excess liquid.
Place filters immediately in the prepared tyramide solution mixture and incubate for 30 min at 37 °C on a shaker (about 75 RMP) in the dark.

Filters should not dry out while transferring into tyramide solution mixture.
Test tyramide solution concentrations between 1 and 3 µl to obtain greatest difference between hybridized cells and background signal.

Washing:

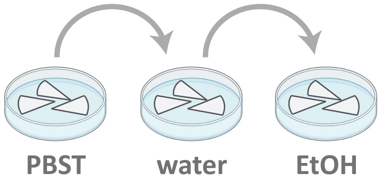

Remove filters from tyramide solution mixture and place them onto blotting paper to remove excess liquid. Put filters directly into PBST for 15 min at RT in the dark. Subsequently, wash filters in deionized sterile water and EtOH for about 1 min each. Let filters dry on a blotting paper in the dark.

Filters should not dry out while transferring into PBST.
► Prolonged incubation in PBST does hardly minimize background fluorescence and is therefore not required.
► If less chlorophyll fluorescence is desired, incubation time in EtOH can be prolonged to 10 min.

DAPI staining:

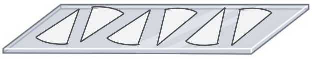

Place dry filters on a glass slide, counterstain with DAPI-Mix (30 µl to 70 µl, depending on the number of filter sections), cover them with a cover glass and store the slides at -20 °C.

All filters sections should be completely covered with DAPI.

Microscopic Analysis:

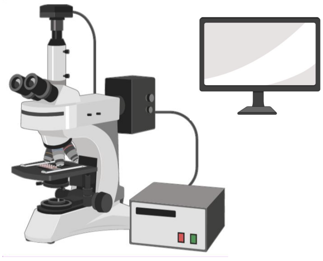

Evaluate filter slides by means of an epifluorescence microscope. To visualize the fluorescence dye FITC, the Zeiss filter set 62 HE (or an equivalent with the same technical properties) should be used.

Make sure that the chosen filter set is compatible with the used fluorescence dye. Wrong filter set can lead to no or a false positive fluorescence signal.

Figure S3: Continued
