# Supplementary figures and images for: FISHing for ciliates: Catalyzed reporter deposition fluorescence in situ hybridization for the detection of planktonic freshwater ciliates
Source: Front Microbiol. 2022 Dec 12;13:1070232. doi: 10.3389/fmicb.2022.1070232 (PMC9790926; doi:10.3389/fmicb.2022.1070232)

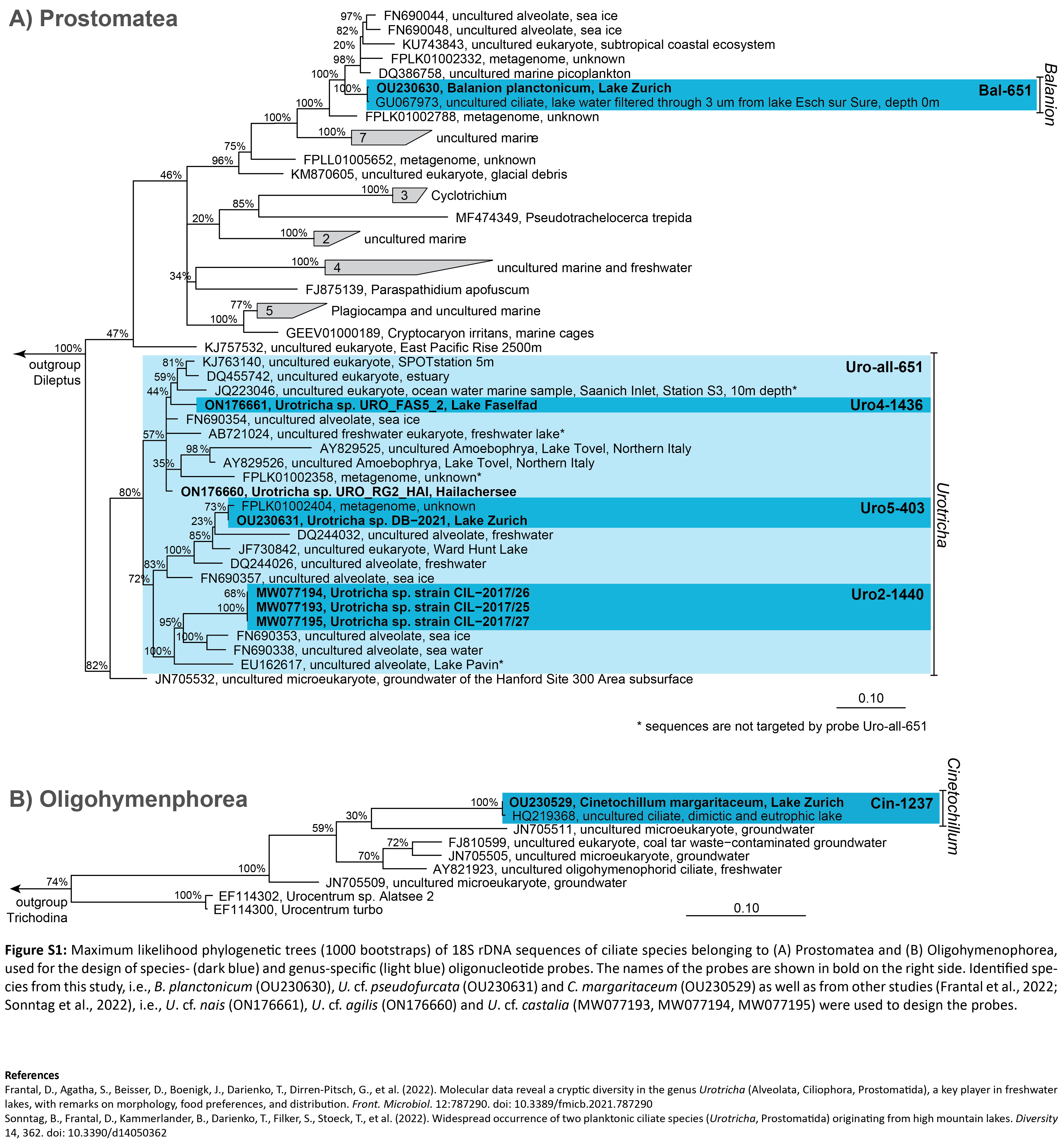

Supplement: Supplementary file 10 [file Image_1.JPEG]

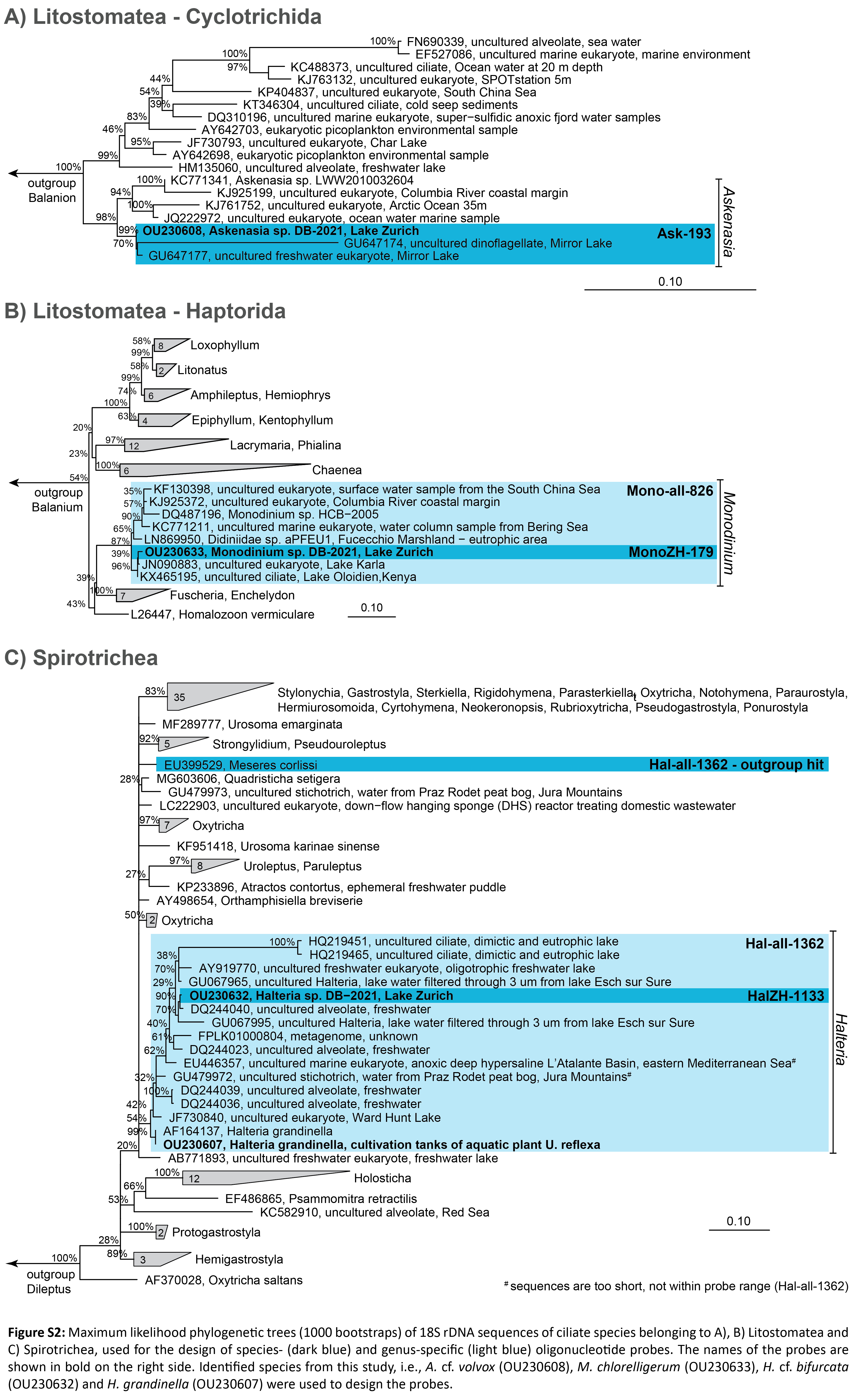

Supplement: Supplementary file 11 [file Image_2.JPEG]

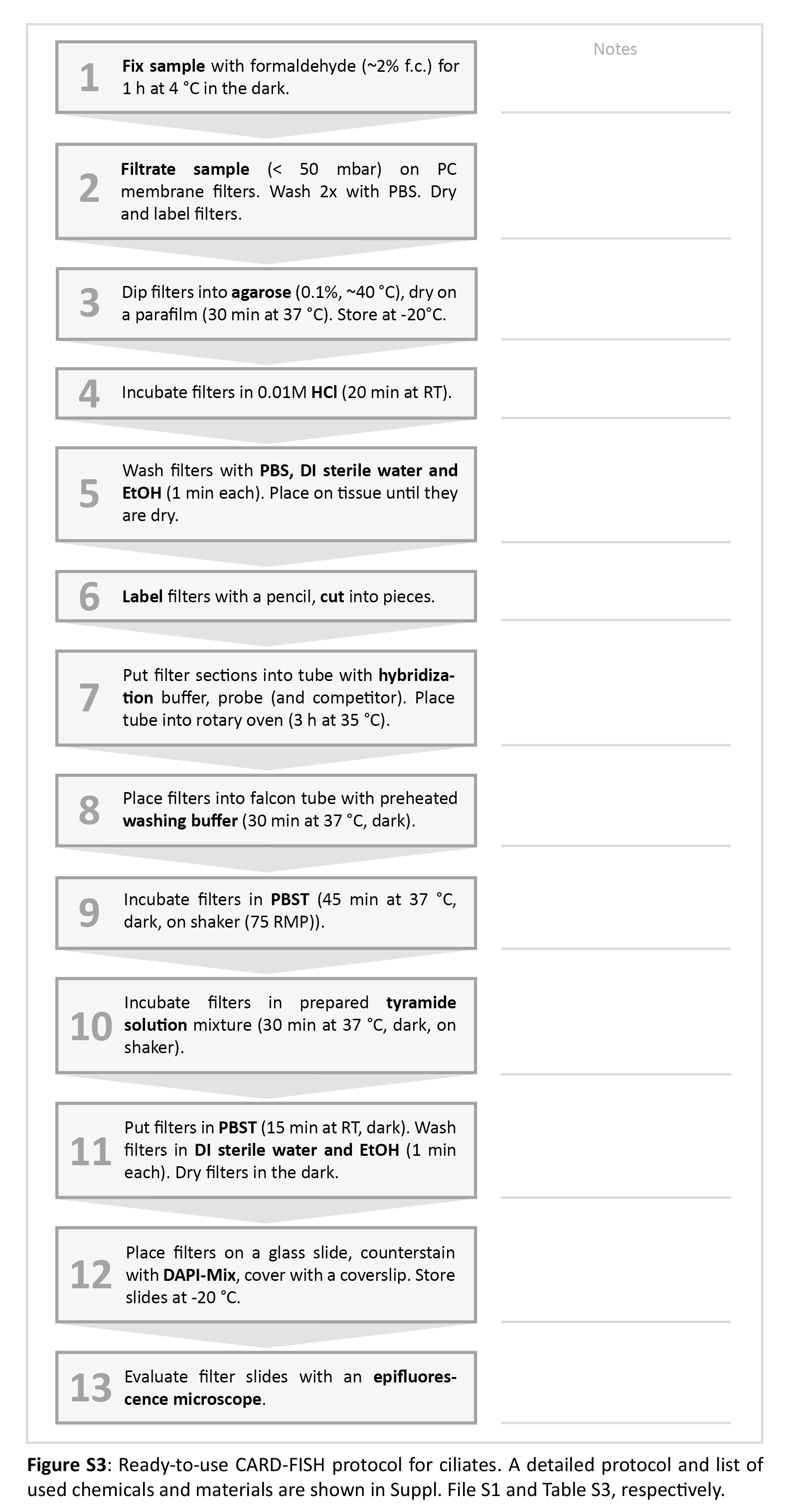

Supplement: Supplementary file 12 [file Image_3.JPEG]

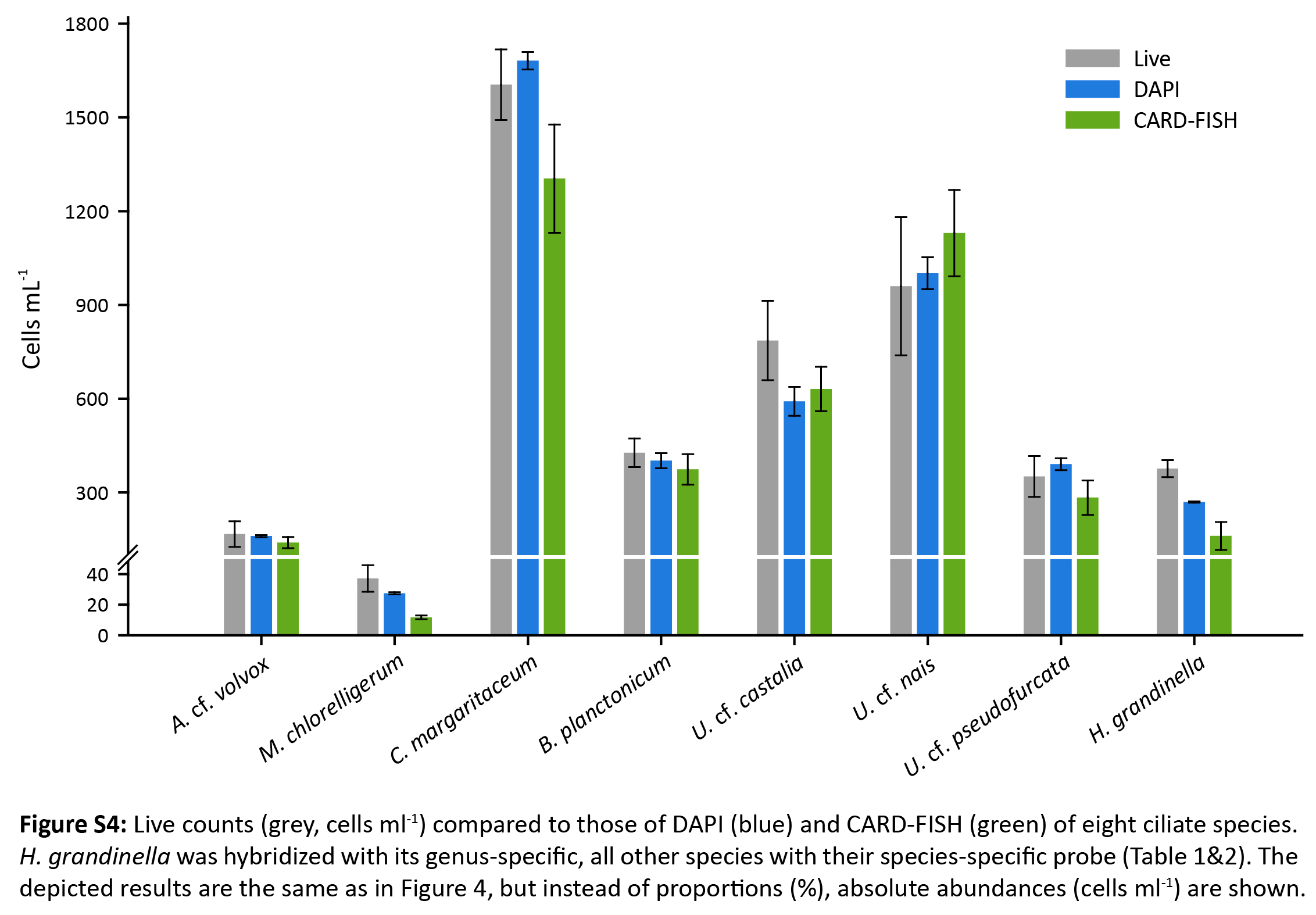

Supplement: Supplementary file 13 [file Image_4.JPEG]

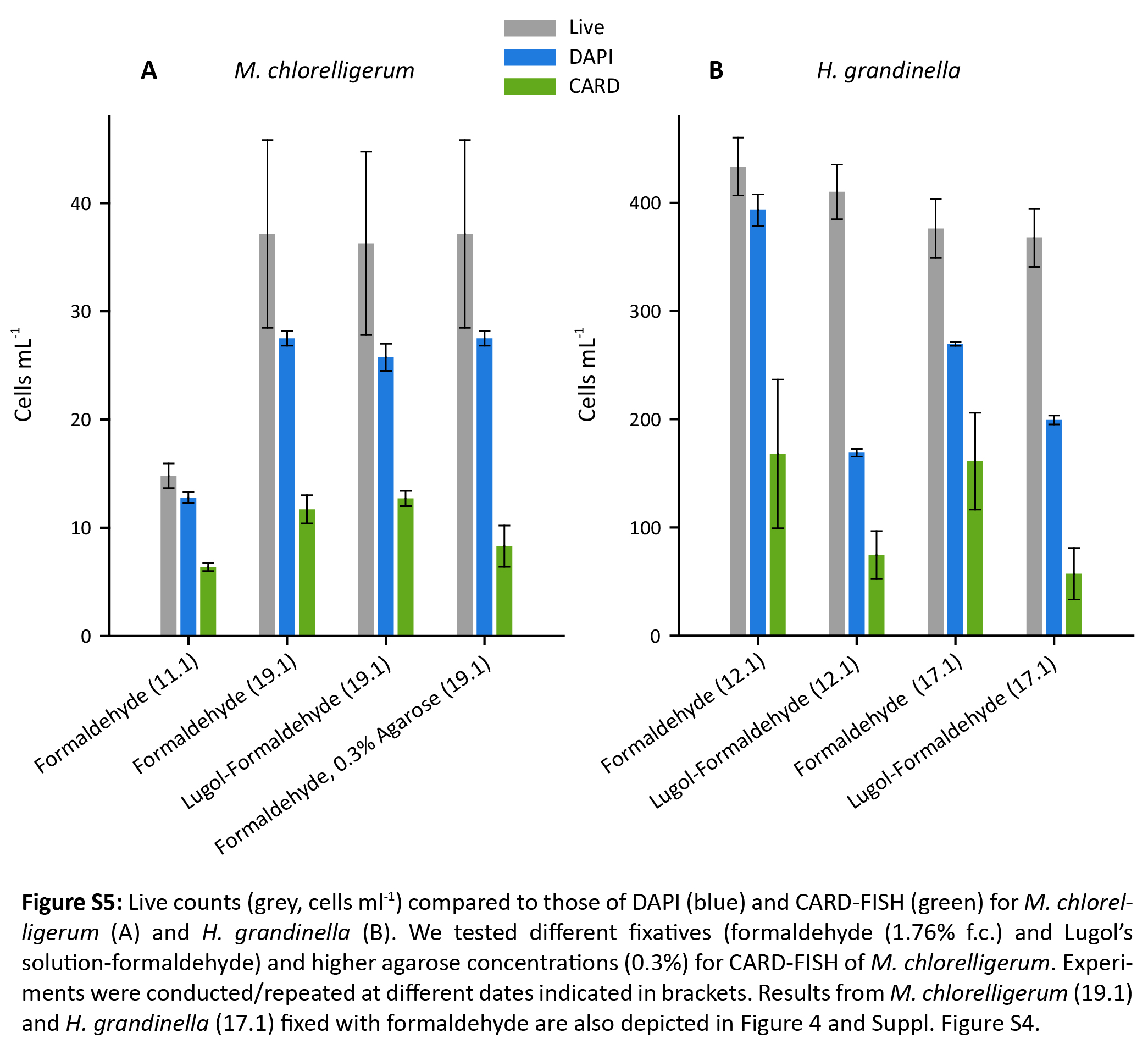

Supplement: Supplementary file 14 [file Image_5.JPEG]
